# Exploring the Binding Mechanism of a Supramolecular Tweezer CLR01 to 14-3-3σ Protein *via* Well-Tempered Metadynamics

**DOI:** 10.3389/fchem.2022.921695

**Published:** 2022-05-12

**Authors:** Xin Zhou, Mingsong Shi, Xin Wang, Dingguo Xu

**Affiliations:** ^1^ College of Chemistry, MOE Key Laboratory of Green Chemistry and Technology, Sichuan University, Chengdu, China; ^2^ State Key Laboratory of Biotherapy/Collaborative Innovation Center of Biotherapy and Cancer Center, West China Hospital, Sichuan University, Chengdu, China; ^3^ Research Center for Material Genome Engineering, Sichuan University, Chengdu, China

**Keywords:** 14-3-3σ, tweezer, CLR01, binding mechanism, supramolecule, well-tempered metadynamics, simulation

## Abstract

Using supramolecules for protein function regulation is an effective strategy in chemical biology and drug discovery. However, due to the presence of multiple binding sites on protein surfaces, protein function regulation *via* selective binding of supramolecules is challenging. Recently, the functions of 14-3-3 proteins, which play an important role in regulating intracellular signaling pathways *via* protein–protein interactions, have been modulated using a supramolecular tweezer, CLR01. However, the binding mechanisms of the tweezer molecule to 14-3-3 proteins are still unclear, which has hindered the development of novel supramolecules targeting the 14-3-3 proteins. Herein, the binding mechanisms of the tweezer to the lysine residues on 14-3-3σ (an isoform in 14-3-3 protein family) were explored by well-tempered metadynamics. The results indicated that the inclusion complex formed between the protein and supramolecule is affected by both kinetic and thermodynamic factors. In particular, simulations confirmed that K214 could form a strong binding complex with the tweezer; the binding free energy was calculated to be −10.5 kcal·mol^−1^ with an association barrier height of 3.7 kcal·mol^−1^. In addition, several other lysine residues on 14-3-3σ were identified as being well-recognized by the tweezer, which agrees with experimental results, although only K214/tweezer was co-crystallized. Additionally, the binding mechanisms of the tweezer to all lysine residues were analyzed by exploring the representative conformations during the formation of the inclusion complex. This could be helpful for the development of new inhibitors based on tweezers with more functions against 14-3-3 proteins *via* modifications of CLR01. We also believe that the proposed computational strategies can be extended to understand the binding mechanism of multi-binding sites proteins with supramolecules and will, thus, be useful toward drug design.

## Introduction

Supramolecular chemistry is a rapidly growing field that has shown broad application prospects in various fields such as sensing, materials science, extraction, and drug delivery (Kolesnichenko and Anslyn, 2017; Williams et al., 2021). Recently, supramolecules have been extensively used to regulate protein functions and demonstrated significant potential for application in drug discovery (Martos et al., 2009; Dang et al., 2013; Yang et al., 2014; van Dun et al., 2017; Finbloom and Francis, 2018; Jagusiak, 2019; Cao et al., 2021). However, improving the binding affinity and selectivity of supramolecules to proteins, which may effectively reduce the dosage and side effects of drugs, is still a challenging task (Reddy and Zhang, 2013; Milroy et al., 2014). The key challenge is that supramolecules can recognize more than one site on the target protein with different binding affinities (Lee et al., 2014; McGovern et al., 2015; Mallon et al., 2016; van Dun et al., 2017; Pan et al., 2018). Moreover, the inhibitors selectively targeting different binding sites may change the signaling pathways of the target protein, thus changing its different function (Milroy et al., 2014; Supuran, 2020). Therefore, it is highly desirable to explore the binding mechanism of supramolecules to multi-sites, which should facilitate the development of new inhibitors with higher binding specificities. Herein, 14-3-3σ, a sub-member belonging to the 14-3-3 proteins, was used as a model protein because there are 17 lysine residues distributed on the protein surface. The protein–protein interactions between 14-3-3σ and its partner proteins have been shown to be regulated by a supramolecular tweezer (CLR01) *via* the formation of inclusion complexes with surface lysine residues (Bier et al., 2013). We, in this work, will try to systematically investigate the association between a tweezer and all surface lysine residues, which may provide theoretical insights into future inhibitor design with high binding specificity.

**FIGURE 1:**
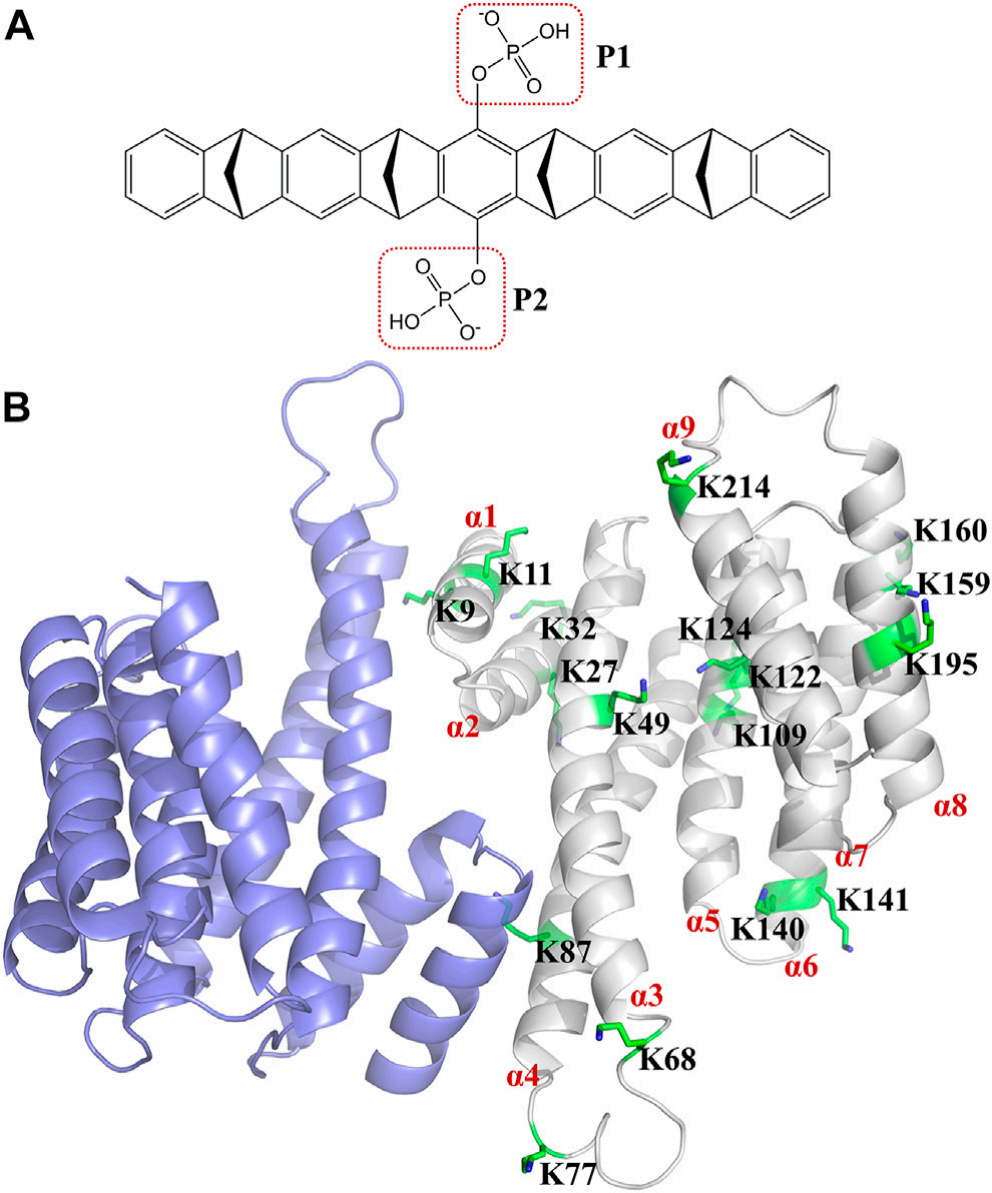
Structure of tweezer (CLR01) and 14-3-3σ. **(A)** shows the structure of the tweezer (CLR01), and **(B)** shows the dimeric structure of 14-3-3σ (Protein Database Bank (PDB) ID: 1YZ5). The α-helix of the 14-3-3σ monomer is labeled in red. The 17 lysine residues on the 14-3-3σ monomer are represented by green sticks, and the detailed location of each lysine residue is depicted in Supplementary Figure S2.

Tweezer CLR01 (CAS number: 1338489-62-5) comprises a belt-shaped open cavity with two rotatable phosphonate groups (Figure 1A) (Talbiersky et al., 2008). It can specifically form more stable inclusion complex with lysine rather than arginine (Fokkens et al., 2005; Talbiersky et al., 2008; Schrader et al., 2016). It has been widely applied to modulate protein functions (Bier et al., 2013; Schrader et al., 2016; Trusch et al., 2016; Vopel et al., 2017; Wilch et al., 2017; Mittal et al., 2018; Li et al., 2019; Bengoa-Vergniory et al., 2020; Weil et al., 2020; Brenner et al., 2021). Additionally, the optimization of CLR01 has gained considerable attention (Dutt et al., 2013a; Dutt et al., 2013b; Klarner and Schrader, 2013; Heid et al., 2018; Herrera-Vaquero et al., 2019; Guillory et al., 2021; Li et al., 2021; Meiners et al., 2021). The 14-3-3 proteins are composed of nine antiparallel α-helices (Yang et al., 2006) and are target proteins for the tweezer (Figure 1B). The 14-3-3 proteins, which include seven human 14-3-3 families (*α/β, ε, η, γ, τ/θ, σ*, and *ζ*), can form homodimers and heterodimers; however, the sigma isoform can only form a homodimer (Wilker et al., 2005; Li et al., 2013). The 14-3-3σ play an important role in biological function in many cancers (Huang et al., 2020; Eum et al., 2021; Winter et al., 2021). The 14-3-3 proteins have hundreds of partner proteins (Yang et al., 2006; Molzan et al., 2010; Schumacher et al., 2010; Herzog et al., 2015; Jia et al., 2019; Park et al., 2019; Falcicchio et al., 2020). For example, 14-3-3 proteins have been employed to locate the partner protein salt-inducible kinase (SIK) in the cytoplasm to regulate the SIK downstream gene (Sonntag et al., 2018). Considering that the protein–protein interactions between the 14-3-3 proteins and their partners can be regulated, these 14-3-3 proteins are potential targets for disease treatment (Aghazadeh and Papadopoulos, 2016; Stevers et al., 2018; Aljabal and Yap, 2020; Winter et al., 2021; Navarrete and Zhou, 2022).

The binding of tweezers to 14-3-3 proteins is a representative example of supramolecules regulating protein functions. Several experimental and theoretical studies have investigated the binding events between tweezers and 14-3-3 proteins (Bier et al., 2013; Bier et al., 2017; Shi and Xu, 2019; Guillory et al., 2021). *Via* isothermal titration calorimetry (ITC), Bier et al. found that tweezers exhibited at least two binding events with different affinities to 14-3-3σ (Bier et al., 2013). However, they discovered a unique binding event for tweezer/K214 (14-3-3σ) *via* crystallization (Supplementary Figure S1) (Bier et al., 2013). Subsequently, the crystal structure of tweezer/K74 (14-3-3ζ) was also determined (Supplementary Figure S1C) (Bier et al., 2017). K74 on 14-3-3ζ has a similar structural environment to K77 on 14-3-3σ (Supplementary Figure S2). Based on these studies, it can be concluded that tweezers can recognize more than one lysine residue of 14-3-3 proteins. Theoretical studies have also confirmed this finding (Bier et al., 2013; Shi and Xu, 2019). The relative quantum mechanical (QM) energies have been evaluated *via* combined quantum mechanical and molecular mechanical (QM/MM) calculations, which indicated that in addition to K214, some other lysine residues (K87, K141, K160, and K195) could also be recognized by the tweezer (Bier et al., 2013). Furthermore, 10 of the 17 lysine residues of 14-3-3σ were identified by the tweezer *via* molecular dynamics (MD) simulations and binding free energy calculations using molecular mechanics/generalized Born surface areas (MM/GBSA) (Shi and Xu, 2019). Recently, based on the tweezer/K214 (14-3-3σ) structure, Guillory et al. fused a tweezer with the partial Exoenzyme S (ExoS) peptide to obtain a highly selective inhibitor (MT-ExoS), which only targeted K214 on 14-3-3σ (Guillory et al., 2021). The highly selective MT-ExoS demonstrated significantly stronger inhibitory affinity than the unmodified tweezer. Nevertheless, the detailed formation process of the inclusion complexes remains elusive, which hinders the development of supramolecules that target the 14-3-3 proteins.

Numerous enhanced sampling methods, such as metadynamics (Laio and Parrinello, 2002), umbrella sampling (Torrie and Valleau, 1977), steered MD (Izrailev et al., 1999), accelerated MD (Hamelberg et al., 2004), and others (Sugita and Okamoto, 1999; Bolhuis et al., 2002; Jorgensen and Thomas, 2008), have been extensively applied to elucidate details of the kinetic effects during substrate binding. Metadynamics is one of the most powerful methods to explore the binding process, including the transition state, pathway of binding/unbinding, conformational rearrangement of the binding site, and the free energy profile of ligand-protein systems (Gervasio et al., 2005; Barducci et al., 2008; Laio and Gervasio, 2008; Dama et al., 2014; Cavalli et al., 2015; Tiwary et al., 2015; Wang et al., 2018; Dodda et al., 2019; Ghosh et al., 2019).

This study employed the well-tempered metadynamics method to understand the recognition mechanism of the surface lysine residues of 14-3-3σ by CLR01. The free energy landscapes for the different binding sites were then evaluated. Finally, the binding mechanisms of the tweezer to the 17 lysine sites on 14-3-3σ were clarified from the kinetic and thermodynamic perspectives. This study can reveal the binding mechanism of the tweezer to the lysine residues of 14-3-3 proteins and provide useful information on the design of novel inhibitors for targeting proteins.

## Computational Details

### System Preparation

The crystal structure of dimeric apo 14-3-3σ [Protein Database Bank (PDB) ID: 1YZ5 (Benzinger et al., 2005)] was obtained from the Protein Database Bank (Berman et al., 2000). Missing residues were constructed using the SWISS-MODEL online service (Waterhouse et al., 2018). The 14-3-3σ protein exists as a homodimer in its active state (Wilker et al., 2005; Verdoodt et al., 2006; Li et al., 2013; Neves et al., 2021). There are 17 lysine residues in human 14-3-3σ [UniProt ID: P31947 (Bateman et al., 2021)]. Among them, five lysine residues (K9, K11, K27, K68, and K87) are located at the interface between the two monomer chains, which may contribute to the formation of the homodimer conformation of 14-3-3σ (Supplementary Figure S3). Therefore, homodimeric 14-3-3σ was selected to create tweezer/14-3-3σ complexes for these five lysine residues. Moreover, monomeric 14-3-3σ was used to construct the initial tweezer/14-3-3σ complexes for the other lysine residues. For these initial tweezer/14-3-3σ complexes, the tweezer was manually moved out of the site from a distance of more than 12 Å to the selected lysine residue (Supplementary Figure S4 shows the initial structures of these complexes).

The force field parameters of CLR01 were generated using the standard general amber force field (Wang et al., 2004) generation procedure. Initially, the geometric structure of the CLR01 molecule was optimized at the B3LYP/6-31G∗ level using Gaussian 09 (Frisch et al., 2009), and the partial atomic charges were determined by the restrained electrostatic potential protocol (Bayly et al., 1993). Then, based on the antechamber program (Wang et al., 2006) implemented in the AMBER18 program, the force field parameters for the tweezer molecules were developed. The Amber ff14SB force field was employed to describe 14-3-3σ (Maier et al., 2015).

The tweezer/14-3-3σ complexes were solvated in rectangular boxes of TIP3P water (Jorgensen et al., 1983) with a margin of 12 Å from the solute in each dimension. Sodium ions were added to these boxes to neutralize the entire system. The sizes of the boxes were approximately 76 Å × 84 Å × 87 Å and 87 Å × 96 Å × 103 Å for monomeric and dimeric 14-3-3σ systems, respectively. Periodic boundary conditions and a nonbonding interaction cut-off of 12 Å were applied. Long-range electrostatic interactions were described by the particle-mesh Ewald algorithm (Darden et al., 1993). The positions of the water molecules were initially relaxed by the steepest descent minimization of 9,000 steps and conjugate gradient minimization of 1,000 steps, wherein all solute molecules were fixed at their original positions. Thereafter, 10,000 steps of conjugate gradient minimization were used to optimize the entire system. Subsequently, the optimized system was gradually heated to 300 K in 100 ps in a constant atom number, volume, and temperature ensemble. Finally, a 500 ps constant atom number, pressure, and temperature equilibrium simulation was conducted at 1 atm and 300 K. All MD simulations were performed using AMBER18 (Case et al., 2018). Thereafter; the last structure was selected as the starting point for metadynamics.

### Well-Tempered Metadynamics

To understand the binding mechanism of the tweezer to these lysine sites on 14-3-3σ, well-tempered metadynamics was performed. Well-tempered metadynamics is an efficient enhanced sampling method to study the binding mechanism of ligands to proteins (Ghosh et al., 2019; Brandt et al., 2021; Wakchaure and Ganguly, 2022). Based on the selected collective variables (CVs), the well-tempered metadynamics method progressively builds up a history-dependent Gaussian-shaped biasing potential (Barducci et al., 2008). The deposited bias potential of well-tempered metadynamics is expressed as

$$V\left(s,t\right)\;=\sum_{t\prime =\tau ,\;2\tau ,\ldots }^{t}\omega e^{-V\left(s,t\prime \right)/\Delta T}exp\langle -\frac{{\left(s-s\left(t\prime \right)\right)}^{2}}{2\sigma^{2}}\rangle \;$$ (1)

where  V(s, t) is the total bias potential added to the system; *t* is the simulation time; s is the CV; t′ represents the deposition time of Gaussian; τ is the deposition time interval for each Gaussian potential; σ is the width of the Gaussian; ω represents the initial height of the Gaussian; and $\omega e^{-V\left(s,t\prime \right)/\Delta T}$ denotes the height of the Gaussian deposited at time t' . With the increase in V(s, t) , $\omega e^{-V\left(s,t\prime \right)/\Delta T}$ decays at virtual temperature ΔT . By rescaling the height of the Gaussians, the convergence of the binding free energy can be achieved at the end of sampling. The free energy surface F(s, t)  can be acquired from  V(s, t) according to the following equation:

$$F\left(s,t\right)\;=\;-\frac{\text{T}+\Delta T}{\Delta T}V\left(s,t\right)$$ (2)

where T is the system temperature.

Two CVs (namely, CV1 and CV2) were selected to improve sampling accuracy. CV1 represents the distance between the center of mass (COM) of the lysine side chain and the COM of the tweezer ring, excluding the two phosphate groups (Supplementary Figure S5). CV2 is the coordination number, defined as the total contact number between the heavy atom in the side chain of lysine and the carbon atom in the tweezer ring. CV2 is modeled as a switching function $\left(C_{N\;}\right)$ :

$$C_{N\;}=\;\sum_{i\in A}\sum_{i\in B}\frac{1-{\left(\frac{r_{ij}}{r_{0}}\right)}^{n}}{1-{\left(\frac{r_{ij}}{r_{0}}\right)}^{m}}$$ (3)

where *A* is the set of heavy atoms of the selected lysine residue on 14-3-3σ; *B* denotes the set of carbon atoms in the tweezer ring within 0.6 nm of any atoms in *A*; $r_{ij}$ is the distance between the atoms i and j ; $r_{0}$ represents the contact distance within which a pair of atoms are considered to be in contact with each other and is set to 0.6 nm. The other parameters use default values.

The widths of the Gaussians for CV1 and CV2 were fixed at 0.05 nm and 2, respectively. Additionally, the initial height of the Gaussian was set to 0.4 kJ mol^−1^, and the deposition time interval for each bias potential was fixed at 0.4 ps with a bias factor of 5. The entire procedure of well-tempered metadynamics was conducted using AMBER18 with PLUMED 2.3.1. (Bonomi et al., 2009). A total of 410 ns metadynamics were performed for tweezer/14-3-3σ models for all 13 systems (See Supplementary Table S1 for details).

## Results and Discussion

### Analysis of Binding Affinity

**FIGURE 2:**
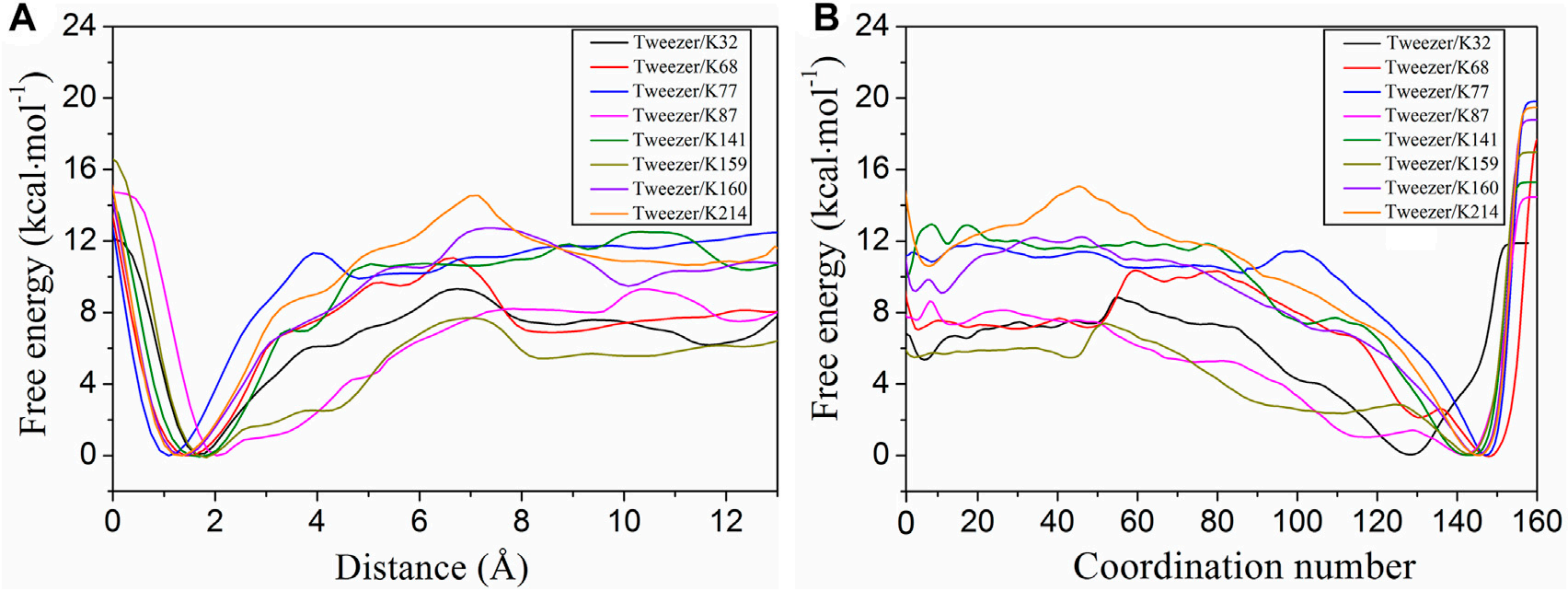
One-dimensional free energy profiles extracted from the two-dimensional free energy surfaces with respect to the distance (CV1) and coordination number (CV2) for the tweezer binding to the eight putative binding sites (K32, K68, K77, K87, K141, K159, K160, and K214). The free energy of the bound state was defined as zero for the tweezer binding to lysine sites. CV1 is the distance between the center of mass (COM) of the lysine side chain and the COM of the tweezer ring, excluding the two phosphate groups. CV2 represents the coordination number between the heavy atom in the side chain of lysine and the carbon atom in the tweezer ring.

Well-tempered metadynamics has been widely used to explore the pathways for the binding or unbinding of ligands to receptors (Dodda et al., 2019; Ghosh et al., 2019; Brandt et al., 2021; Pang et al., 2022). Well-tempered metadynamics was used to calculate the two-dimensional free energy landscapes for the binding of the tweezer to all surface lysine residues (Supplementary Figure S6). To further elucidate the binding process along the two CVs, we plotted the one-dimensional free energy curves using CV1 and CV2as the binding coordinates, respectively (Figure 2). The convergence of the binding free energies calculations for different models was examined, and all systems were found to have converged (Supplementary Figure S7). The free energies of the bound states were scaled to zero for the tweezer/14-3-3σ complexes. Thus, a negative value of the binding free energy indicates the formation of a stable host–guest inclusion complex. Combining the computed free energies with the simulation trajectories, the tweezer/lysine bound state was found to occur when CV1 was less than 2.5 Å and CV2 was approximately 125.0.

According to the evolution of CV1 and CV2 with respect to the simulation time (Supplementary Figure S8), only 8 of the 17 lysine residues can form stable inclusion complexes with the tweezer in the simulation time. These eight lysine residues had different binding affinities to the tweezer, and the binding affinities of K77, K141, K160, and K214 to the tweezer were significantly higher than those of K32, K68, K87, and K159 (Figure 2; Supplementary Table S2). Therefore, based on the binding states and affinities of these 17 lysine sites to the tweezer, these sites were divided into three types: strong binding sites, moderate binding sites, and unbinding sites. The strong binding sites (K77, K141, K160, and K214) were bound to the tweezer and had strong binding free energies. The tweezer recognized the moderate binding sites (K32, K68, K87, and K159) with weaker binding affinity than the strong binding sites. The remaining nine sites (K9, K11, K27, K49, K109, K121, K122, K140, and K195) were classified as unbinding sites, which could not be stably bound with the tweezer during the simulation time. Thus, we will focus on those lysine residues belonging to strong and moderate binding sites in this section.

From the binding free energy profiles, the tweezer/K214 complex has the lowest binding free energy of −10.5 kcal mol^−1^. The binding of the tweezer with K77, K141, and K160 results in relatively stable inclusion complexes with slightly lower binding free energies of −10.1, −10.3, and −9.3 kcal mol^−1^, respectively. Previous QM/MM calculations have also indicated that K214, K141, and K160 are strong binding sites (Bier et al., 2013). Furthermore, K74 on 14-3-3ζ has been reported to form an inclusion complex with the tweezer from the crystal structure (PDB ID: 5M37) (Bier et al., 2017). As 14-3-3 proteins are a highly conserved family of proteins (Benzinger et al., 2005; Babula and Liu, 2015), the environment of K77 on 14-3-3σ is quite similar to that of K74 on 14-3-3ζ (Supplementary Figure S2 for details). Therefore, K77 on 14-3-3σ has substantial potential to be recognized by the tweezer. Moreover, the energy barriers for tweezer binding to K214, K77, K141, and K160 were 3.7, 1.3, 2.6, and 3.2 kcal mol^−1^, respectively (Figure 2; Supplementary Table S2). The low energy barriers for these strong binding sites indicated that the association of the tweezer and 14-3-3σ would be rapid, which was consistent with the binding kinetics acquired from the surface plasmon resonance analysis of the tweezer binding to immobilized 14-3-3σ (Bier et al., 2013). Considering the relatively small difference between the calculated binding free energies by metadynamics, it is difficult to identify which lysine residue (K77, K141, K160, or K214) was bound with the tweezer. Therefore, we may simply classify these four sites as belonging to a single class.

In addition to the strong binding sites, the moderate binding sites are also important. The binding free energies of K32, K68, K87, and K159 are −5.7, −7.0, −7.5, and −5.5 kcal mol^−1^, respectively (Figure 2). The results indicate that the moderate binding site can still be well-bound to the tweezer, albeit with a weaker binding affinity than K77, K141, K160, and K214. Furthermore, the energy barriers for K32, K68, K87, and K159 are 3.3, 3.8, 1.8, and 2.1 kcal mol^−1^, respectively. The low energy barriers for these sites also indicate that their association with the tweezer is rapid, consistent with the finding obtained for the strong binding sites. Similar to the strong binding sites, the small differences in the binding free energies and association barrier heights make it hard to distinguish between these four sites. More importantly, we have the calculated binding affinity range for the CLR01 to all surface lysine residues on 14-3-3σ to be between −5.5 and −10.5 kcal mol^−1^, which is in good agreement with the experimental measurements of −6.2 ± 0.5 and −8.9 ± 0.1 kcal mol^−1^ (Bier et al., 2013). Moreover, according to Bier et al. (2013), there are most likely two binding events for the CLR01 to 14-3-3σ based on ITC measurements of the binding free energies. It should be pointed out that ITC cannot identify which sites are specifically bound. Two putative binding events based on the binding free energy calculation can be partially established in our simulation. Our simulations are thus consistent with experimental observation. Of course, we must emphasize that the binding process in real samples might simultaneously involve multiple lysine residues.

The relative QM energy for each tweezer/lysine inclusion complex only considered the bound states in the previous QM/MM calculations. It cannot offer the enough information for the thermodynamic and kinetic properties of tweezer binding to lysine sites. It is interesting to compare the binding free energies obtained by metadynamics and the MM/GBSA approach. Previously, the MM/GBSA method was employed to calculate the binding free energies for the binding of the tweezer to the lysine sites on 14-3-3σ based on the classical MD simulation. The corresponding results were included in Table 1 for comparison (Shi and Xu, 2019). First of all, both methods suggested that more than one lysine residue could be well-recognized by the supramolecular CLR01, which is consistent with experimental observation. However, the quantitative differences in these binding free energies between the two methods were significant. For example, the binding free energy was calculated to be approximately −2.5 kcal mol^−1^ for the K214 site by MM/GBSA, while a value of −10.5 kcal mol^−1^ was obtained by metadynamics. The result obtained by metadynamics may be more reasonable as the crystal structure was obtained from the tweezer co-crystallized with K214. According to Table 1, opposite recognition results were observed for the binding of K27, K87, K109, and K195 to the tweezer. Because K87 is located at the interface between the two monomer chains, it was simply neglected in the previous classical MD simulations (Shi and Xu, 2019). In contrast, metadynamics revealed that the cavity formed by the two monomer chains provided accessible space for the binding of the tweezer to K87.

**TABLE 1:** Comparison between the binding free energies calculated by metadynamics and molecular mechanics/generalized Born surface area (MM/GBSA) method. Values are given as the absolute values for all binding free energies. Units are in kcal·mol^−1^.

Type	Lysine site	Binding energy barrier	ΔG		
			Metad.^a^	MM/GBSA^b^	QM/MM^c^
Strong binding sites	K214	3.7	−10.5	−2.5	24.3
	K77	1.3	−10.1	−12.8	—
	K141	2.6	−10.3	−12.8	0
	K160	3.2	−9.3	−3.8	22.6
Moderate binding sites	K32	3.3	−5.7	−9.3	—
	K68	3.8	−7.0	−4.7	54.4
	K87	1.8	−7.5	—	22.0
	K159	2.1	−5.5	−9.3	39.3
Unbinding sites	K9	—	—	—	70.8
	K11	—	—	—	47.7
	K27	—	—	−12.8	45.9
	K49	—	—	—	56.2
	K109	—	—	−6.6	40.3
	K122	—	—	—	—
	K124	—	—	—	75.1
	K140	—	—	—	57.0
	K195	—	—	−14.3	26.9

a Metad. denotes an abbreviation for metadynamics.

b Binding free energy calculated from MM/GBSA method (Shi and Xu, 2019).

c The relative quantum mechanical (QM) energy was calculated from QM/MM method as respect to the top binding site (K141) (Bier et al., 2013).

*Experimental binding free energies for tweezer binding with 14-3-3σ was −6.2 ± 0.5 and −8.9 ± 0.1 kcal·mol−^1^ (Bier et al., 2013).

Of course, in some cases, the MM/GBSA method performs well, e.g., the interactions of oligosaccharides to carbohydrate-binding module families (Li et al., 2018; Wang and Xu, 2019). However, it has been well understood that the MM/GBSA method overestimates the charge interactions (Zhu et al., 2007; Guimaraes and Mathiowetz, 2010; Ravindranathan et al., 2011; Mikulskis et al., 2012). For the tweezer/lysine complex system, the tweezer has two phosphate groups with a negative charge, and the lysine residue has a positive charge. This implies that the MM/GBSA method may overestimate the charge-charge interactions and thus cause a large deviation between the calculated and experimental binding free energies. Additionally, the binding of any substrate to proteins should consider two aspects, i.e., thermodynamic and kinetic factors (Camilloni and Pietrucci, 2018; c; 2020; Lyu et al., 2020; Zhang et al., 2020; Fu et al., 2022). The MM/GBSA method can only reflect the effects from the thermodynamic perspective, which might also result in some deviations in describing the substrate binding. The difference between the results obtained by classical MD simulations and metadynamics indicates that both thermodynamic and kinetic processes must be considered to rationally calculate the binding free energies and explore the binding mechanisms for ligands to receptors, at least for the tweezer/14-3-3σ system investigated in this work.

### Conformational Analysis of Binding

According to the binding free energy calculations presented above, CLR01 can form inclusion complexes with multiple lysine residues on 14-3-3σ without specificity. To improve the recognition selectivity of CLR01 with lysine residues distributed in different surface positions on 14-3-3σ protein, the conformational change information along the binding coordinates needs to be further analyzed. The free energy landscapes for the tweezer binding with different lysine sites offer a general overview of the kinetic processes of the binding events. To reveal the details of the binding process and the influence of the environments of the lysine sites on protein surfaces, representative conformations of the unbound state, transition state, and bound state were utilized to explore the kinetic process of tweezer binding to these 17 lysine sites.

K214, K77, K141, and K160 are located at different sites in the active pocket of 14-3-3σ. Structurally, K214, K77, and K141 are primarily situated at the edge of the protein active cavity, whereas K160 is located at the back of the active cavity (Figure 1B, Supplementary Figure S3). Additionally, K214, K77, K141, and K160 are located in the terminal regions of the helix (α9, α4, α6, and α6, respectively), which is near the loop region. Moreover, the number of residues close to (less than 4 Å away) K214, K77, K141, and K160 are 6, 6, 8, and 7, respectively. In brief, these strong binding sites are situated in the terminal region of the α-helix, and there are few other residues around these lysine residues.

**FIGURE 3:**
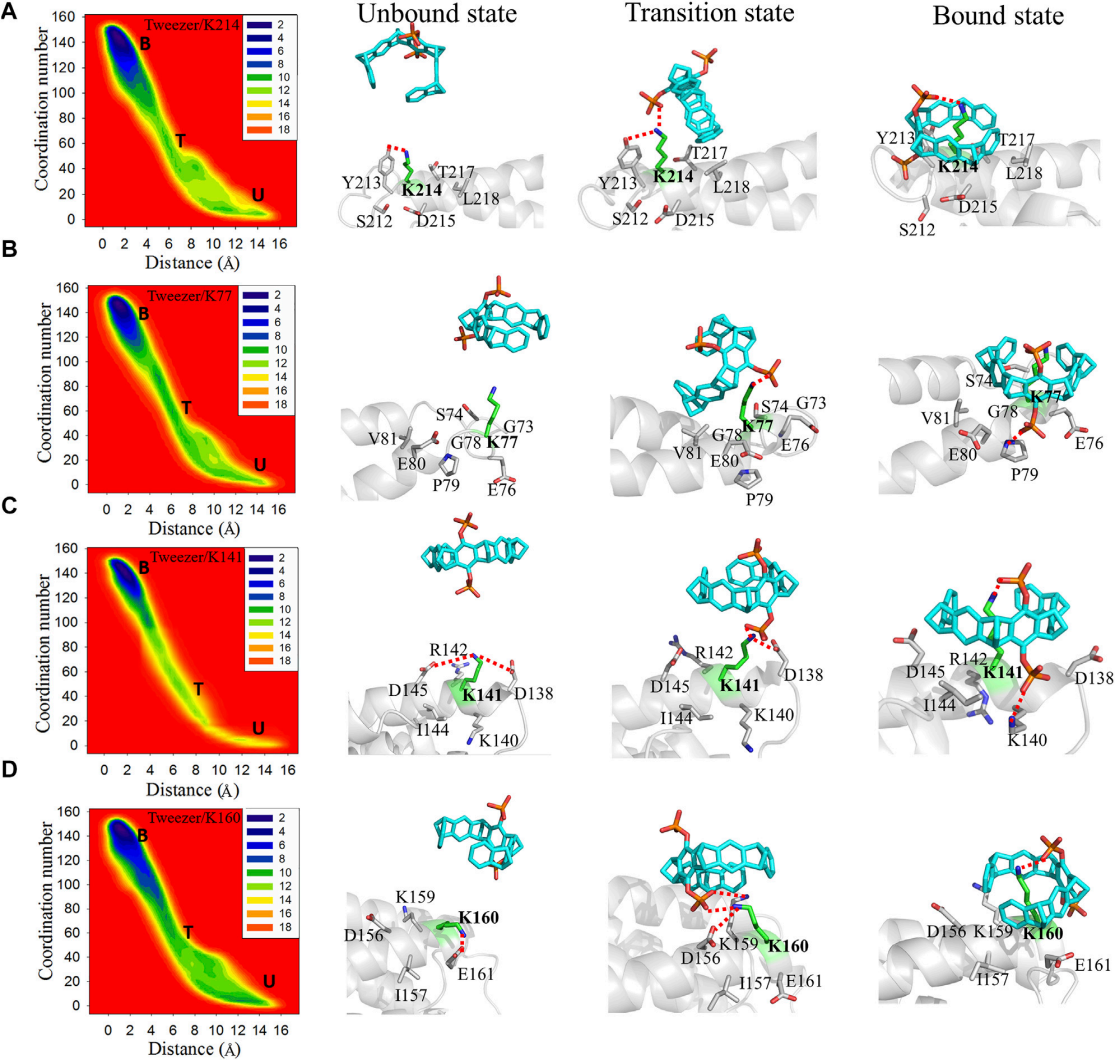
Two-dimensional free energy landscapes and representative structures for the binding of the tweezer to **(A)** K214, **(B)** K77, **(C)** K141, and **(D)** K160 on 14-3-3σ. The symbols “U,” “T,” and “B” in the free energy landscapes indicate the unbound, transition, and bound states, respectively. The representative structures of the unbound states, transition states, and bound states of these lysine residues to the tweezer are shown on the right side of the free energy landscapes. The superimposed structures of the three states for each binding site are depicted in Supplementary Figure S9.

For apo-14-3-3σ, Y213, situated in the terminal region α9 around K214, can form a stable hydrogen bond with K214 (Figure 3A). In the transition state, the interaction between K214 and Y213 is disrupted. Furthermore, the down phosphate group (P2) of the tweezer develops a new electrostatic interaction with the side chain of K214. Subsequently, the tweezer forms an inclusion complex with K214 *via* the rotation of its ring. The phosphate group (P1) of the tweezer produces a stable electrostatic interaction with the ammonium ion of lysine (D_N-P_ = 4.21 ± 0.77 Å). Additionally, a hydrophobic interaction develops between the nonpolar regions of Y213, L218, and T217 and the hydrophobic ring of CLR01, which is a key factor in stabilizing the bound state. The binding model for tweezer/K214 is also consistent with the corresponding crystal structures (PDB IDs: 5OEH and 5OEG) (Bier et al., 2013).

The side chain of K77 remains fully exposed to the solvate environment in apo 14-3-3σ. Moreover, G73 and G78 around K77 have short side chains that cannot affect K77 (Figure 3B, Supplementary Figure S10). Therefore, the side chain of K77 can be expected to be highly flexible. These environmental residues around K77 provide spatially accessible sites for tweezer binding, which results in a small energy barrier to tweezer binding (1.3 kcal mol^−1^). The phosphate group of the tweezer interacts electrostatically with the ammonium ion of K77 without disrupting the extra nonbonding interactions (Figure 3B). In the bound state, the hydroxyl groups in the phosphate group form electrostatic interactions with P79. Furthermore, the alkyl groups of E76, E80, and V81 establish additional hydrophobic interactions with the aliphatic ring of the tweezer, which stabilize the tweezer/K77 inclusion complex.

In the unbound state, K141 can interact with D145 and D138 *via* electrostatic interactions. Additionally, K141 lies adjacent to positive residues such as R142 and K140, which are staggered and do not interfere with each other in apo 14-3-3σ. These interacting residues have long side chains, which can be rearranged to generate inclusion complexes between the tweezer and K141 (Supplementary Figure S9). In the transition state, the tweezer rotates such that its phosphate group destroys the electrostatic interaction between K141 and D138. In the bound state (tweezer/K141), the phosphate group produces an additional strong salt bridge with the ammonium ion of K140 *via* the rotation of the tweezer. This leads to a more stable inclusion complex and a lower binding free energy.

**FIGURE 4:**
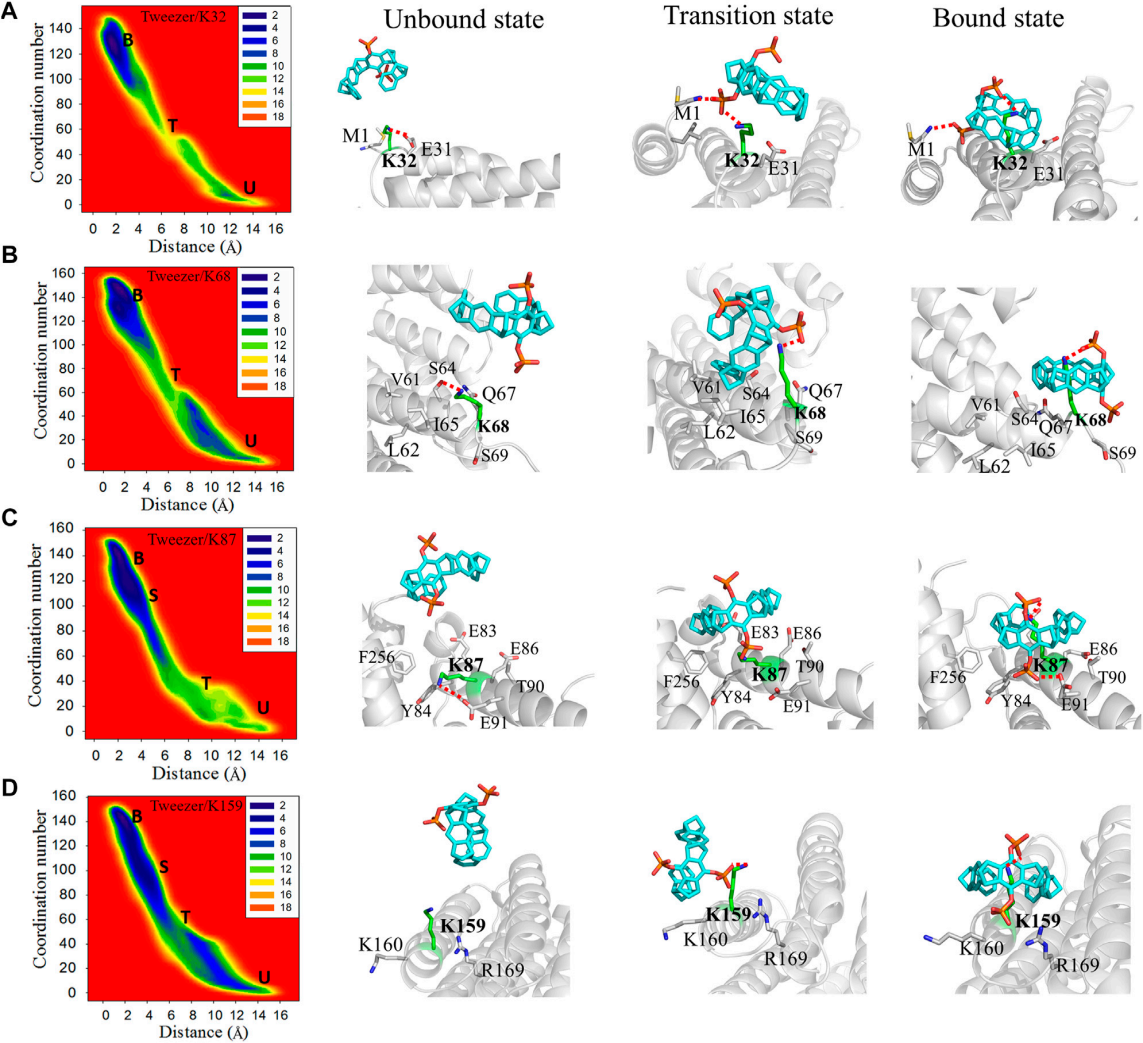
Two-dimensional free energy landscapes and representative structures for the binding of the tweezer to **(A)** K32, **(B)** K68, **(C)** K87, and **(D)** K159. The symbols “U”, “T”, “S”, and “B” in the free energy landscapes represent the unbound, transition, semi-bound, and bound states, respectively. Additionally, the representative structures of the unbound state, transition state, and bound state for the tweezer to these lysine residues are shown on the right side of the free energy landscapes. The superimposed structures of these states for each binding site are depicted in Supplementary Figure S12.

K160 can form electrostatic interactions with E161 and D156 in apo 14-3-3σ (Figure 3D, Supplementary Figure S11). In the transition state, the side chains of D156 and E161 with negative charges can generate electrostatic repulsion with a negative charge of a phosphate group, which causes the transition of the tweezer far away from E161. Furthermore, the phosphate group can interact with K159 and K160. To avoid repulsion between the phosphate group and E161 in the bound state, the tweezer sharply flipped to promote the movement of the phosphate group toward the solvent. Additionally, no interaction was observed between the amino group of K159 and the phosphate group, which was ascribed to the mismatch between the accessible space and the molecular size of the tweezer (Supplementary Figure S11). Thus, during the formation of the bound state (Figure 4D), sufficient stable geometric space must be available to accommodate the tweezer. An extra hydrophobic interaction can form between I157 and K159 with the tweezer ring.

Strong binding sites have three common features. First, the sites located at the end of the helix and close to the loop region afford enough space to accommodate the phosphate group of the tweezer. Second, no negatively charged residues, including acidic residues, exist around the phosphate group of the tweezer. Third, the hydrophobic residues around these lysine sites can contribute to the binding affinities of these sites to the tweezer by forming hydrophobic interactions with the tweezer ring.

The moderate binding sites (K32, K68, and K159) are located at the ends of α2, α3, and α6, respectively. Simultaneously, K87 is situated in the middle of α4 and points to the interface between the two monomer chains. This arrangement is similar to that of the strong binding sites, which are also located at the terminals of the helix other than K87. The numbers of residues around the moderate binding sites (10, 6, 10, and 10 for K32, K68, K87, and K159, respectively) are higher than those around the strong binding sites (6, 6, 8, and 7 for K214, K77, K141, and K160, respectively). This indicates that the moderate binding sites have more crowded spatial environments than those of the strong binding sites.

K32, located close to the N-terminus of the α1 helix, can form a weak electrostatic interaction with E31 in the apo 14-3-3σ (Figure 4A). The tweezer has to overcome not only the electrostatic interactions between K32 and E31 but also the attraction between the ammonium ion of M1 and the tweezer from the unbound model to the bound model. In the transition state, the phosphate group establishes an electrostatic interaction with M1 and K32. In the bound state, M1 contributes to the stability of the tweezer/K32 inclusion complex by forming electrostatic interactions with other phosphate groups of the tweezer. Moreover, no additional factor stabilizes the tweezer/K32 inclusion complex, resulting in weaker binding free energy than the strong binding sites. K68 can form electrostatic interaction with S64 in the unbound state (Figure 4B). Furthermore, several hydrophobic residues, such as V60, L61, and I65, were noticed around K68 and composed a hydrophobic region in 14-3-3σ. In the transition state, in addition to the electrostatic interaction between the phosphate group and K68, the hydrophobic region near K68 interacts with the tweezer ring to increase the difficulty of tweezer binding. Therefore, it suggests a higher energy barrier for this lysine site. Phosphate group P2 pointed to the solvent in the bound state, and phosphate group P1 formed electrostatic interaction with K68. In addition, no other significant interaction is present to stabilize the tweezer/K68 inclusion complex.

Initially, an electrostatic interaction formed between E91 and K87 can be observed in the apo 14-3-3σ (Figure 4C). However, the phosphate group P2 replaced the E91 to form an electrostatic interaction with K87 at the transition state. The hydrophobic environment, centered on K87 (F256, Y84, E86, and T90), attracted the tweezer to form a semi-bound state for K87 (Supplementary Figure S13). The distance between tweezer and K87 is 3.45 ± 0.67 Å for this semi-bound state (Supplementary Figure S14). The side chain of K87 entered the cavity of the tweezer from the twisting conformation and formed a tightly bound state, which can be found from the smaller CV1 value of 2.09 ± 0.43 Å. Phosphate group P2 can be orientated toward the cavity at the interface between the two monomer chains of 14-3-3σ in the bound state. In addition, T90, F256, and Y84 can stabilize the tweezer/K87 complex through the hydrophobic interactions with the tweezer. Although the rearrangement of the side chain of K87 can be observed in the simulations, the space around K87 is sufficient to accommodate the tweezer. Meanwhile, the hydrophobic environment contributes to stabilizing the final tweezer/K87 complex.

K159 is sandwiched between the basic amino acid K160 and R169 in the unbound state (Figure 4D). Thus, the roles of K160 and R169 cannot be ignored when the tweezer binds with this site. The phosphate group of CLR01 can establish an electrostatic interaction with K159. The tweezer tends to stay between K159 and K160 with little hydrophobic interaction between the aliphatic ring of the tweezer and the nonpolar parts of K159 or K160 in the transition state. In the semi-bound state, the phosphate group of CLR01 forms an electrostatic interaction with R169 (Supplementary Figure S12). Meanwhile, the interaction between the tweezer and K159 is weaker than the bound state according to the values of CV1, i.e., 3.61 ± 0.64 vs. 2.46 ± 0.97 Å for semi-bound and bound states, respectively (Figure 4D, Supplementary Figure S14). When the tweezer forms the bound state with K159, phosphate group P2 is pointed toward the solvent environment, and phosphate group P1 mainly forms an electrostatic interaction with the side chain of K159.

The moderate binding sites can form an inclusion complex with tweezers from the accessible spatial environments. However, the residues around the lysine sites can interfere with the binding of tweezer to lysines through conformation rearrangement. In fact, such kind of binding competition could result in decreasing the binding affinity or increasing the binding energy barrier, e.g., the E31 competed with phosphate group of CLR01 for K32 site; the R169 competed with K159 for K159 site; the hydrophobic environment (V60, L61, and I65) competed with aromatic ring of CLR01 for K68 site; the E91 competed with phosphate group of CLR01 for K87 site. This indicates that moderate binding affinity can be obtained for those binding sites in relation to that for the strong binding sites, which agrees with binding free energy calculations.

Some lysine residues (K9, K11, K27, K49, K109, K122, K124, K140, and K195) are not easily bound by the tweezer due to their particular topological environments on the 14-3-3σ. Obvious steric hindrances can be observed around K9, K122, K124, and K140 (Supplementary Figure S15). K9 and K124, having similar orientations, are located in the middle of α1 and α5, respectively, which are deeply trapped in their adjacent α-helices to hinder tweezer binding. K122 is in the bottom of the active cavity, which is composed of α5 and is adjacent to α3 and α7, and cannot be appropriately enclosed by the tweezer. Furthermore, K140, situated at the terminal of the α6 helix and pointing to the α5 helix, has a significant steric hindrance, preventing the binding of the tweezer to it. Therefore, their associations with the tweezer were not considered in this study. Moreover, the bindings of K11, K27, K49, K109, and K195 to the tweezer are unfavorable and different from other sites (Supplementary Figure S16). This also can be found from simulations in this work with the evolutions of CV1 and CV2 along the simulation time for these lysine sites (Supplementary Figure S8).

The active cavity of 14-3-3 protein is used to recognize partner proteins. Some small inhibitors and stabilizers modulate the 14-3-3 protein function by targeting this active cavity (Zhao et al., 2011; Iralde-Lorente et al., 2019; Gigante et al., 2020). Theoretically, K11 and K49, which are deeply located in the active pocket, can be bound to inhibit the activity of 14-3-3 σ. Nevertheless, K11 and K49 cannot be bound by tweezers due to steric hindrance. For example, K11 is situated in the middle of the α1 helix and is close to the α2 helix. Although the tweezer entered the amphiphilic active cavity of 14-3-3σ, the binding of the tweezer to K11 did not occur in our simulations (Supplementary Figure S16). This further proves that if there is insufficient space to accommodate the phosphate group of the tweezer, the lysine site in the middle of the helix on the protein cannot be recognized by the tweezer. The simulations implied that in the case of K49, the tweezer generally stayed in the active cavity and had a very short time to make contact with K49 (Supplementary Figure S16). Thus, the tweezer probably interacts with amphiphilic cavities rather than with the lysine sites in the active cavity.

E31, T98, L102, and H106, located on the α2 and α3 helices, are close to K27 to hinder the tweezer binding. Additionally, the electrostatic interaction between E31 and K27 means that the side chain of K27 cannot be recognized by the tweezer (Supplementary Figure S16). The number of residues (distance less than 4 Å from K27) also was calculated as 12. It indicates that the steric hindrance around K27 is the main reason it remains unbound by the tweezer.

**FIGURE 5:**
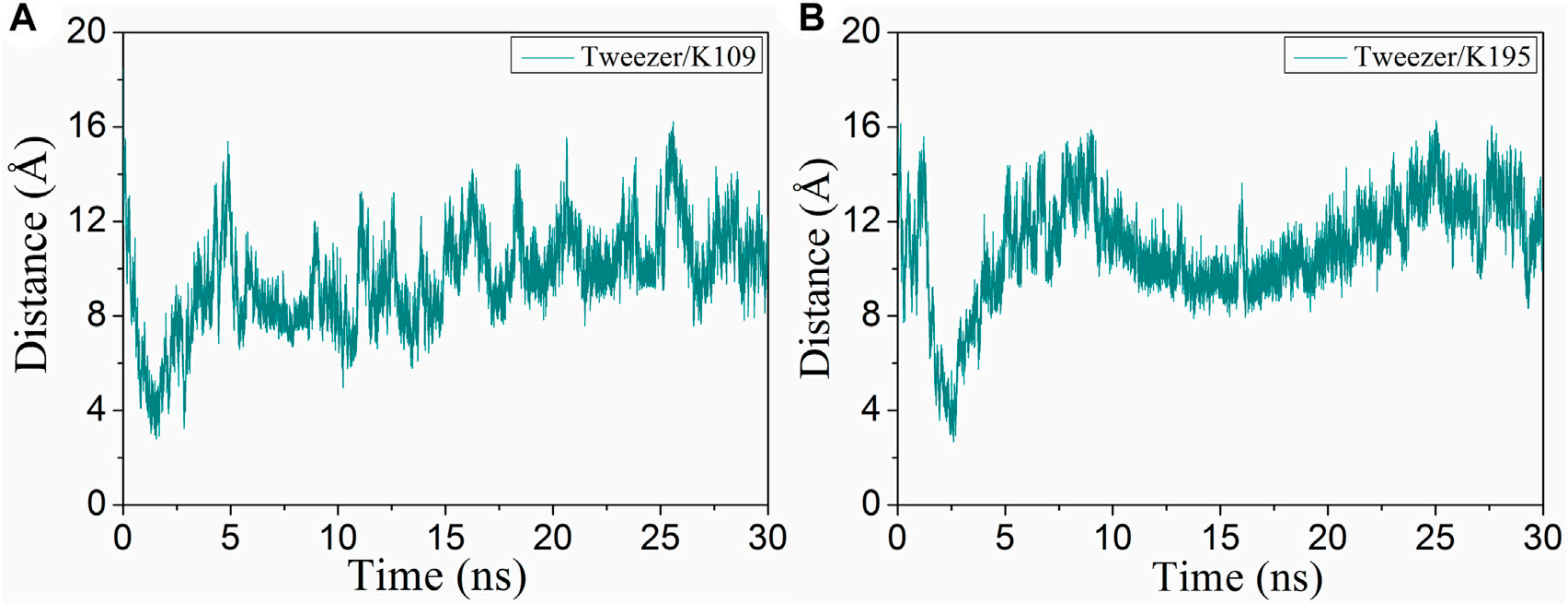
Evolutions of the distance (CV1) of the binding of the tweezer to **(A)** K109 and **(B)** K195 with respect to the simulation time.

K109 is located at the back of the active cavity and is near the loop region. During the simulation, the tweezer/K109 inclusion complex could not be stably formed mainly owing to the repulsion between E110 and the phosphate group of the tweezer (Figure 5). The tweezer is consistently attracted to the circular hydrophobic cavity: its aromatic ring establishes hydrophobic interactions with the nonpolar parts of L121, R117, K109, and I108; its phosphate groups form electrostatic interactions with K124, R117 or R117, and K109 (Supplementary Figure S17). Therefore, it can be speculated that if there is a suitable hydrophobic environment and electrostatic attraction near the lysine site, the tweezer will prefer to remain in this hydrophobic environment and produce electrostatic interactions with the positively charged amino acids rather than forming an inclusion complex with the lysine residue.

K195 is situated in the middle of α8, and the tweezer only forms a transient semi-inclusion complex with it at 2.6 ns (Figure 5B). The following aspects can explain this observation. First, there is not enough space to accommodate the phosphate group of CLR01 due to the presence of L227, R224, and F198 on the one side of the α8. Second, located on the other side of α8, S192, T196, and D200 hinder the phosphate group through electrostatic repulsion. Third, D199 and I191 also prevent CLR01 binding with K195 by steric hindrance (Supplementary Figure S18). Thus, both steric hindrance and electrostatic repulsion result in non-binding between CLR01 and K195.

For the unbinding sites, one general feature is that the large steric hindrances prevent the tweezer binding with lysine residues and further block the formation of inclusion complexes with lysine residues. As we described above, for those lysine sites located in the middle of the helix or pointing toward the inside of 14-3-3σ, the lysine sites show large steric hindrances and insufficient space to accommodate the tweezer. Therefore, the steric hindrance determines tweezer binding or non-binding inclusion complexes with lysine residues on 14-3-3σ.

### Binding Mechanism of CRL01 to Lysine Sites

In this work, based on the detailed characterization of the recognition processes of tweezer binding with lysine sites from the metadynamics calculations, the detailed mechanistic proposals between lysine residues on 14-3-3σ and tweezer can be derived. The formation of an inclusion complex between CLR01 and lysine is mainly determined by the effect of steric hindrance. If we carefully examine the residues distributed around the lysine residues, we can find the sources of the spatial hindrance. As we can see, when the residue around the binding site features a long and flexible side chain, which is located at the interface between two secondary structure domains or toward the inside of the protein, no stable inclusion complex could be formed. Indeed, due to significant steric hindrance, the tweezer cannot bind with the K9, K11, K27, K49, K122, K124, K195, and K140 (Supplementary Figure S19).

**FIGURE 6:**
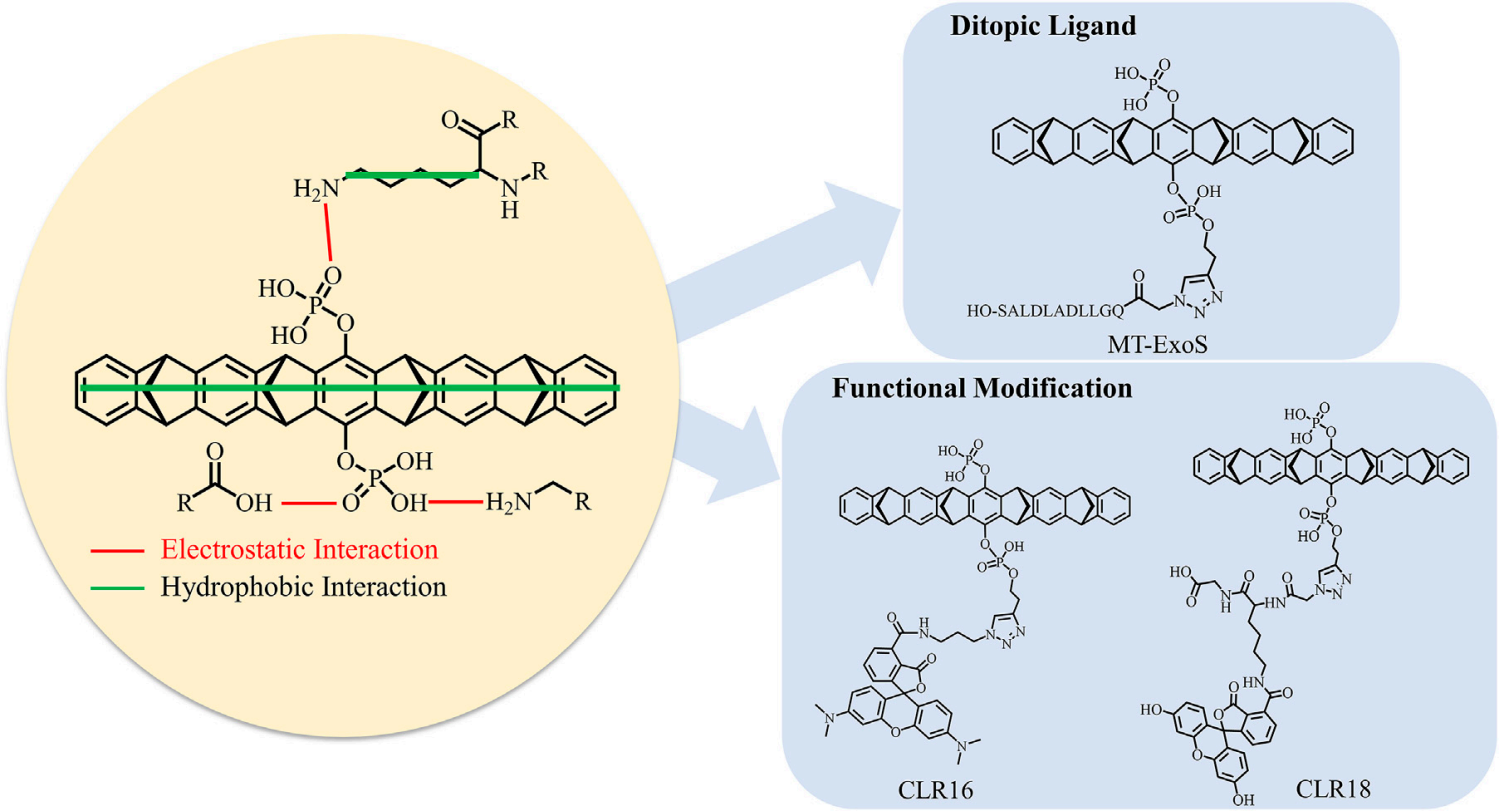
Binding mechanism of tweezer CLR01 binding to lysine site on 14-3-3σ and the optimization strategies of tweezer.

On the other hand, negatively charged residues around the lysine residues will compete with the phosphate groups of the tweezer and thus increase the binding energy barrier. For example, the electrostatic interaction between K160 and E161 is unfavorable for CLR01 binding with K160 and increases the energy barrier to 3.2 kcal mol^−1^ (Supplementary Figure S11). Electrostatic interactions for K141 (K141-D145, K141-D138), K160 (K160-E161, K160-D156), K32 (K32-E31), K68 (E68-S64) also result in increasing the energy barrier heights to 2.6, 3.2, 3.3, 3.8 kcal mol^−1^, respectively. Those interactions found in the apo 14-3-3σ can increase the energy barrier for CLR01 binding with lysine on the 14-3-3σ surface. In a word, due to recognition competition provided by the protein itself, negatively charged residues around the lysine may have the chance to form electrostatic interaction with the side chain of lysine to compete with the tweezer, thus increasing the association energy barrier. Of course, we should emphasize that such structural features might not affect the final formation of the inclusion complex. In addition, the supramolecules prefer recognizing side chains, peptide motifs, and a specific protein context of the target proteins (Urbach and Ramalingam, 2011; McGovern et al., 2012; McGovern et al., 2015; van Dun et al., 2017). For the case of 14-3-3σ, the inclusion complex of CLR01/lysine is mainly stabilized by the hydrophobic interactions between the cavity of the tweezer and the aliphatic hydrocarbon chains of lysine residues (Figure 6). Additional stabilization factors can be attributed to the electrostatic interactions between the phosphate groups and the positively charged ammonium group of lysine at the binding sites. For example, the phosphonate group of CLR01 can form a hydrogen bond with P79 in K77/tweezer complex (Supplementary Figure S20).

Our calculations and previous experimental characterization show that a non-specific recognition style can be fully established to bind the supramolecule CLR01 to target lysine residues on 14-3-3σ. Our simulations can observe two binding events consistent with experimental measurements. Hence, we can develop new inhibitors with special functions using just those lysine residues as anchor points. In this way, the relative position of the active regions of 14-3-3σ to positions of strong and moderate binding sites might be the most important issue in the development of the ditopic ligands via the supramolecular inclusion complex (Supplementary Figure S21). Therefore, correctly identifying the preferred binding sites would still be a necessary step. In the current work, the free energy landscapes for the association of the tweezer with different lysine residues provide such an opportunity. According to our simulations, the main contribution to the stability of the CLR01/14-3-3σ inclusion complexes came from the hydrophobic interactions between the cavity of the tweezer and the aliphatic hydrocarbon chains of lysine residues. It indicates that the benzene and norbornadiene rings of the CLR01 are ideally situated for stabilizing the side chain of lysine and should be retained for inhibitor optimization in the future. Meanwhile, additional stabilization is provided by the interaction between one phosphate group of CLR01 with the ammonium group of lysine (Figure 6). Some researchers have suggested that the other phosphate group can be modified to achieve some special functions, such as regulating the protein–protein interaction. For example, recent work by Guillory et al. (2021) indicates that linking the ExoS peptide to the phosphate group of CLR01 (labeled as MT-ExoS and shown in Figure 6) results in a site-selective binding with K214 on 14-3-3σ. More importantly, the MT-ExoS is a ditopic ligand that can regulate the PPI against the ExoS peptide-binding into the active site of 14-3-3σ. The K214 stays at the edge of the active site; one of the phosphate groups points toward the active space, which leaves the position available for future inhibitor design to obtain better effects against possible PPIs. Interestingly, the ExoS peptide and CLR01 units can find their unique binding sites on the 14-3-3 protein surface (Supplementary Figure S22). Meanwhile, the binding affinity of MT-ExoS (K_D_ = 0.41 µM) to 14-3-3σ was improved in relation to CLR01 (K_D_ = 8.39 µM) and ExoS (K_D_ = 45.60 µM) as determined by ITC. Therefore, we may expect that new ditopic molecules can be designed to have some special functions with higher binding. Indeed, trials in this direction have been reported. Very recently, the fluorescent groups 5-carboxytetramethylrhodamine (CLR16) and 6-fluorescein amidite (CLR18) were added to the phosphate group of the CLR01 to increase the intrinsic fluorescence for exploring its cell penetrance and intracellular distribution (Figure 6) (Li et al., 2021). NMR and fluorescence titrations with Ac-Lys-OMe were performed to find that CLR16 (K_D_ = 1.3 nM) and CLR18 (K_D_ = 3.7 nM) bind lysine in a similar manner to CLR01 and with stronger affinity than CLR01 (K_D_ = 20 µM) (Talbiersky et al., 2008).

## Conclusion

In this study, the binding mechanism of a supramolecular tweezer CLR01 to the lysine sites on 14-3-3σ was revealed *via* metadynamics. Among the 17 lysine sites on 14-3-3σ, 8 sites with different affinities to the tweezer can form inclusion complexes with the tweezer. Through the analysis of the representative conformations during the tweezer binding to the lysine sites, the differences between the thermodynamic and kinetic results were revealed, and the characteristics of the recognized lysine sites were summarized. The spatial hindrance around the lysine sites was the dominant factor in determining whether or not the tweezer/lysine inclusion complex formed. This is reasonable considering the belt-shaped topology of the tweezer molecule.

In addition, according to binding free energies, we can classify these eight sites into two classes—the strong and moderate binding sites. Free energy profiles of the formation of putative inclusion complexes imply that these sites have accessible binding energy barriers ranging from 1.3 to 3.8 kcal mol^−1^. K214, with the strongest binding free energy and reasonable binding energy barrier, indicates that it is a stable binding site easily accessible to the tweezer for binding, which may partially explain the co-crystallization of the tweezer at the K214 position. Moreover, the average binding free energy calculated for the strong binding sites (K77, K141, K160, and K214) is −10.0 ± 0.5 kcal mol^−1^, while it is −6.4 ± 1.0 kcal mol^−1^ for the moderate binding sites (K32, K68, K87, and K159). Our simulations are in good agreement with experimental ITC measurements that identify two binding events, in which the binding free energies were estimated at −6.2 ± 0.5 kcal mol^−1^ and −8.9 ± 0.1 kcal mol^−1^. It should be emphasized that current simulations confirmed that the tweezer binds to the surface lysine residues of 14-3-3σ in a non-specific way. More importantly, due to the similar binding affinities in the two classes of binding sites, it is difficult to distinguish these lysine sites from each other when the binding event occurs. Such kind of binding characteristics then defines the possible direction of developing new inhibitors to regulate the function of 14-3-3σ based on the tweezer framework or other supramolecules. In summary, these lysine residues that favor the recognition should be considered as anchor points, while major modifications should be made to the phosphate group of the tweezer molecule. This would result in ditopic ligands to inhibit different functions of 14-3-3σ. Finally, we believe that the computational strategy to identify the binding sites should be particularly useful for those proteins containing multiple binding sites and is, thus, helpful for future inhibitor design.

## Data Availability

The original contributions presented in the study are included in the article/Supplementary Material, further inquiries can be directed to the corresponding authors.

## References

[B1] Aghazadeh Y. Papadopoulos V. (2016). The Role of the 14-3-3 Protein Family in Health, Disease, and Drug Development. Drug Discov. Today 21, 278–287. 10.1016/j.drudis.2015.09.012 26456530

[B2] Aljabal G. Yap B. K. (2020). 14-3-3σ and its Modulators in Cancer. Pharmaceuticals 13, 441. 10.3390/ph13120441 PMC7761676 33287252

[B3] Babula J. J. Liu J.-Y. (2015). Integrate Omics Data and Molecular Dynamics Simulations toward Better Understanding of Human 14-3-3 Interactomes and Better Drugs for Cancer Therapy. J. Genet. Genomics 42, 531–547. 10.1016/j.jgg.2015.09.002 26554908

[B4] Barducci A. Bussi G. Parrinello M. (2008). Well-tempered Metadynamics: a Smoothly Converging and Tunable Free-Energy Method. Phys. Rev. Lett. 100, 020603. 10.1103/PhysRevLett.100.020603 18232845

[B5] Bateman A. Martin M. J. Orchard S. Magrane M. Agivetova R. Ahmad S. (2021). Uniprot: the Universal Protein Knowledgebase in 2021. Nucleic Acids Res. 49, D480–D489. 10.1093/nar/gkaa1100 33237286 PMC7778908

[B6] Bayly C. I. Cieplak P. Cornell W. Kollman P. A. (1993). A Well-Behaved Electrostatic Potential Based Method Using Charge Restraints for Deriving Atomic Charges: the RESP Model. J. Phys. Chem. 97, 10269–10280. 10.1021/j100142a004

[B7] Bengoa-Vergniory N. Faggiani E. Ramos-Gonzalez P. Kirkiz E. Connor-Robson N. Brown L. V. (2020). CLR01 Protects Dopaminergic Neurons *In Vitro* and in Mouse Models of Parkinson's Disease. Nat. Commun. 11, 4885. 10.1038/s41467-020-18689-x 32985503 PMC7522721

[B8] Benzinger A. Popowicz G. M. Joy J. K. Majumdar S. Holak T. A. Hermeking H. (2005). The Crystal Structure of the Non-liganded 14-3-3σ Protein: Insights into Determinants of Isoform Specific Ligand Binding and Dimerization. Cell Res. 15, 219–227. 10.1038/sj.cr.7290290 15857576

[B9] Berman H. M. Westbrook J. Feng Z. Gilliland G. Bhat T. N. Weissig H. (2000). The Protein Data Bank. Nucleic Acids Res. 28, 235–242. 10.1093/nar/28.1.235 10592235 PMC102472

[B10] Bier D. Mittal S. Bravo-Rodriguez K. Sowislok A. Guillory X. Briels J. (2017). The Molecular Tweezer CLR01 Stabilizes a Disordered Protein-Protein Interface. J. Am. Chem. Soc. 139, 16256–16263. 10.1021/jacs.7b07939 29039919 PMC5691318

[B11] Bier D. Rose R. Bravo-Rodriguez K. Bartel M. Ramirez-Anguita J. M. Dutt S. (2013). Molecular Tweezers Modulate 14-3-3 Protein-Protein Interactions. Nat. Chem. 5, 234–239. 10.1038/nchem.1570 23422566

[B12] Bolhuis P. G. Chandler D. Dellago C. Geissler P. L. (2002). Transition Path Sampling: Throwing Ropes over Rough Mountain Passes, in the Dark. Annu. Rev. Phys. Chem. 53, 291–318. 10.1146/annurev.physchem.53.082301.113146 11972010

[B13] Bonomi M. Branduardi D. Bussi G. Camilloni C. Provasi D. Raiteri P. (2009). Plumed: a Portable Plugin for Free-Energy Calculations with Molecular Dynamics. Comput. Phys. Commun. 180, 1961–1972. 10.1016/j.cpc.2009.05.011

[B14] Brandt A. A. M. L. Rodrigues-da-Silva R. N. Lima-Junior J. C. Alves C. R. de Souza-Silva F. (2021). Combining Well-Tempered Metadynamics Simulation and SPR Assays to Characterize the Binding Mechanism of the Universal T-Lymphocyte Tetanus Toxin Epitope TT830-843. BioMed Res. Int. 2021, 1–15. 10.1155/2021/5568980 34285916 PMC8275407

[B15] Brenner S. Braun B. Read C. Weil T. Walther P. Schrader T. (2021). The Molecular Tweezer CLR01 Inhibits Antibody-Resistant Cell-To-Cell Spread of Human Cytomegalovirus. Viruses 13, 1685. 10.3390/v13091685 34578265 PMC8472163

[B16] Camilloni C. Pietrucci F. (2018). Advanced Simulation Techniques for the Thermodynamic and Kinetic Characterization of Biological Systems. Adv. Phys. X 3, 1477531. 10.1080/23746149.2018.1477531

[B17] Cao W. Qin X. Wang Y. Dai Z. Dai X. Wang H. (2021). A General Supramolecular Approach to Regulate Protein Functions by Cucurbit[7]uril and Unnatural Amino Acid Recognition. Angew. Chem. Int. Ed. 60, 11196–11200. 10.1002/anie.202100916 33580548

[B18] Case D. A. Ben-Shalom I. Y. Brozell S. R. Cerutti D. S. Cheatham I. Cruzeiro V. W. D. (2018). Amber 2018. San Francisco: University of California.

[B19] Cavalli A. Spitaleri A. Saladino G. Gervasio F. L. (2015). Investigating Drug-Target Association and Dissociation Mechanisms Using Metadynamics-Based Algorithms. Acc. Chem. Res. 48, 277–285. 10.1021/ar500356n 25496113

[B20] Dama J. F. Parrinello M. Voth G. A. (2014). Well-tempered Metadynamics Converges Asymptotically. Phys. Rev. Lett. 112, 240602. 10.1103/PhysRevLett.112.240602 24996077

[B21] Dang D. T. Nguyen H. D. Merkx M. Brunsveld L. (2013). Supramolecular Control of Enzyme Activity through Cucurbit[8]uril-Mediated Dimerization. Angew. Chem. Int. Ed. 52, 2915–2919. 10.1002/anie.201208239 23355250

[B22] Darden T. York D. Pedersen L. (1993). Particle Mesh Ewald: AnN⋅Log(N) Method for Ewald Sums in Large Systems. J. Chem. Phys. 98, 10089–10092. 10.1063/1.464397

[B23] Dodda L. S. Tirado-Rives J. Jorgensen W. L. (2019). Unbinding Dynamics of Non-nucleoside Inhibitors from HIV-1 Reverse Transcriptase. J. Phys. Chem. B 123, 1741–1748. 10.1021/acs.jpcb.8b10341 30571126 PMC6395492

[B24] Dutt S. Wilch C. Gersthagen T. Talbiersky P. Bravo-Rodriguez K. Hanni M. (2013a). Molecular Tweezers with Varying Anions: a Comparative Study. J. Org. Chem. 78, 6721–6734. 10.1021/jo4009673 23750919

[B25] Dutt S. Wilch C. Gersthagen T. Wölper C. Sowislok A. A. Klärner F.-G. (2013b). Linker Effects on Amino Acid and Peptide Recognition by Molecular Tweezers. Eur. J. Org. Chem. 2013, 7705–7714. 10.1002/ejoc.201301211

[B26] Eum W. S. Kim D. W. Yeo E. J. Yeo H. J. Choi Y. J. Cha H. J. (2021). Transduced Tat-PRAS40 Prevents Dopaminergic Neuronal Cell Death through ROS Inhibition and Interaction with 14-3-3σ Protein. Free Radic. Biol. Med. 172, 418–429. 10.1016/j.freeradbiomed.2021.06.026 34175438

[B27] Falcicchio M. Ward J. A. Macip S. Doveston R. G. (2020). Regulation of P53 by the 14-3-3 Protein Interaction Network: New Opportunities for Drug Discovery in Cancer. Cell Death Discov. 6, 126. 10.1038/s41420-020-00362-3 33298896 PMC7669891

[B28] Finbloom J. A. Francis M. B. (2018). Supramolecular Strategies for Protein Immobilization and Modification. Curr. Opin. Chem. Biol. 46, 91–98. 10.1016/j.cbpa.2018.05.023 30041103

[B29] Fokkens M. Schrader T. Klärner F.-G. (2005). A Molecular Tweezer for Lysine and Arginine. J. Am. Chem. Soc. 127, 14415–14421. 10.1021/ja052806a 16218636

[B30] Frisch M. J. Trucks G. W. Schlegel H. B. Scuseria G. E. Robb M. A. Cheeseman J. R. (2009). Gaussian 09 Rev. A01. Wallingford, CT.

[B31] Fu T. Zheng Q. Zhang H. (2022). Investigation of the Molecular and Mechanistic Basis for the Regioselective Metabolism of Midazolam by Cytochrome P450 3A4. Phys. Chem. Chem. Phys. 24, 8104–8112. 10.1039/d2cp00232a 35319551

[B32] Gervasio F. L. Laio A. Parrinello M. (2005). Flexible Docking in Solution Using Metadynamics. J. Am. Chem. Soc. 127, 2600–2607. 10.1021/ja0445950 15725015

[B33] Ghosh S. Jana K. Ganguly B. (2019). Revealing the Mechanistic Pathway of Cholinergic Inhibition of Alzheimer's Disease by Donepezil: a Metadynamics Simulation Study. Phys. Chem. Chem. Phys. 21, 13578–13589. 10.1039/c9cp02613d 31173012

[B34] Gigante A. Sijbesma E. Sánchez‐Murcia P. A. Hu X. Bier D. Bäcker S. (2020). A Supramolecular Stabilizer of the 14‐3‐3ζ/ERα Protein‐Protein Interaction with a Synergistic Mode of Action. Angew. Chem. Int. Ed. 59, 5284–5287. 10.1002/anie.201914517 PMC7155037 31814236

[B35] Guillory X. Hadrović I. de Vink P. J. Sowislok A. Brunsveld L. Schrader T. (2021). Supramolecular Enhancement of a Natural 14-3-3 Protein Ligand. J. Am. Chem. Soc. 143, 13495–13500. 10.1021/jacs.1c07095 34427424

[B36] Guimarães C. R. W. Mathiowetz A. M. (2010). Addressing Limitations with the MM-GB/SA Scoring Procedure Using the Watermap Method and Free Energy Perturbation Calculations. J. Chem. Inf. Model. 50, 547–559. 10.1021/ci900497d 20235592

[B37] Hamelberg D. Mongan J. McCammon J. A. (2004). Accelerated Molecular Dynamics: a Promising and Efficient Simulation Method for Biomolecules. J. Chem. Phys. 120, 11919–11929. 10.1063/1.1755656 15268227

[B38] Heid C. Sowislok A. Schaller T. Niemeyer F. Klärner F.-G. Schrader T. (2018). Molecular Tweezers with Additional Recognition Sites. Chem. Eur. J. 24, 11332–11343. 10.1002/chem.201801508 30015416

[B39] Herrera-Vaquero M. Bouquio D. Kallab M. Biggs K. Nair G. Ochoa J. (2019). The Molecular Tweezer CLR01 Reduces Aggregated, Pathologic, and Seeding-Competent α-synuclein in Experimental Multiple System Atrophy. Biochimica Biophysica Acta (BBA) - Mol. Basis Dis. 1865, 165513. 10.1016/j.bbadis.2019.07.007 PMC8425273 31319154

[B40] Herzog G. Shmueli M. D. Levy L. Engel L. Gazit E. Klärner F.-G. (2015). The Lys-specific Molecular Tweezer, CLR01, Modulates Aggregation of the Mutant P53 DNA Binding Domain and Inhibits its Toxicity. Biochemistry 54, 3729–3738. 10.1021/bi501092p 26030124

[B41] Huang Y. Yang M. Huang W. (2020). 14-3-3 σ: A Potential Biomolecule for Cancer Therapy. Clin. Chim. Acta 511, 50–58. 10.1016/j.cca.2020.09.009 32950519

[B42] Iralde-Lorente L. Cau Y. Clementi L. Franci L. Tassone G. Valensin D. (2019). Chemically Stable Inhibitors of 14-3-3 Protein-Protein Interactions Derived from BV02. J. Enzyme Inhibition Med. Chem. 34, 657–664. 10.1080/14756366.2019.1574779 PMC8853708 30727786

[B43] Izrailev S. Stepaniants S. Isralewitz B. Kosztin D. Lu H. Molnar F. (1999). Steered Molecular Dynamics. Lect. Notes Comput. Sci. Eng. 4, 39–65. 10.1007/978-3-642-58360-5_2

[B44] Jagusiak A. (2019). An Outline of the Use of Supramolecular Compounds in Biology and Medicine. Acta Biochim. Pol. 66, 545–549. 10.18388/abp.2019_2910 31883361

[B45] Jia Y. Li H.-Y. Wang J. Wang Y. Zhang P. Ma N. (2019). Phosphorylation of 14-3-3ζ Links YAP Transcriptional Activation to Hypoxic Glycolysis for Tumorigenesis. Oncogenesis 8, 31. 10.1038/s41389-019-0143-1 31076568 PMC6510816

[B46] Jorgensen W. L. Chandrasekhar J. Madura J. D. Impey R. W. Klein M. L. (1983). Comparison of Simple Potential Functions for Simulating Liquid Water. J. Chem. Phys. 79, 926–935. 10.1063/1.445869

[B47] Jorgensen W. L. Thomas L. L. (2008). Perspective on Free-Energy Perturbation Calculations for Chemical Equilibria. J. Chem. Theory Comput. 4, 869–876. 10.1021/ct800011m 19936324 PMC2779535

[B48] Klärner F.-G. Schrader T. (2013). Aromatic Interactions by Molecular Tweezers and Clips in Chemical and Biological Systems. Acc. Chem. Res. 46, 967–978. 10.1021/ar300061c 22725723

[B49] Kolesnichenko I. V. Anslyn E. V. (2017). Practical Applications of Supramolecular Chemistry. Chem. Soc. Rev. 46, 2385–2390. 10.1039/c7cs00078b 28317053

[B50] Laio A. Gervasio F. L. (2008). Metadynamics: a Method to Simulate Rare Events and Reconstruct the Free Energy in Biophysics, Chemistry and Material Science. Rep. Prog. Phys. 71, 126601. 10.1088/0034-4885/71/12/126601

[B51] Laio A. Parrinello M. (2002). Escaping Free-Energy Minima. Proc. Natl. Acad. Sci. U.S.A. 99, 12562–12566. 10.1073/pnas.202427399 12271136 PMC130499

[B52] Lee C.-C. Maestre-Reyna M. Hsu K.-C. Wang H.-C. Liu C.-I. Jeng W.-Y. (2014). Crowning Proteins: Modulating the Protein Surface Properties Using Crown Ethers. Angew. Chem. Int. Ed. 53, 13054–13058. 10.1002/anie.201405664 PMC4288931 25287606

[B53] Li L. Erwin N. Möbitz S. Niemeyer F. Schrader T. Winter R. H. A. (2019). Dissociation of the Signaling Protein K‐Ras4B from Lipid Membranes Induced by a Molecular Tweezer. Chem. Eur. J. 25, 9827–9833. 10.1002/chem.201901861 31141233

[B54] Li P. Zhang C. Xu D. (2018). Molecular Dynamics Investigations of Cello-Oligosaccharide Recognition by Cel9G-CBM3c from clostridium Cellulovorans. Phys. Chem. Chem. Phys. 20, 5235–5245. 10.1039/c7cp07175b 29399685

[B55] Li Z. Peng H. Qin L. Qi J. Zuo X. Liu J.-Y. (2013). Determinants of 14-3-3σ Protein Dimerization and Function in Drug and Radiation Resistance. J. Biol. Chem. 288, 31447–31457. 10.1074/jbc.M113.467753 24043626 PMC3814741

[B56] Li Z. Siddique I. Hadrović I. Kirupakaran A. Li J. Zhang Y. (2021). Lysine-selective Molecular Tweezers Are Cell Penetrant and Concentrate in Lysosomes. Commun. Biol. 4, 1076. 10.1038/s42003-021-02603-2 34521989 PMC8440717

[B57] Limongelli V. (2020). Ligand Binding Free Energy and Kinetics Calculation in 2020. Wires Comput. Mol. Sci. 10, e1455. 10.1002/wcms.1455

[B58] Lyu N. Wang K. Zhang F. Qin H. Zhao Y. Wu R. (2020). Recognition of PDL1/L2 by Different Induced-Fit Mechanisms of PD1: a Comparative Study of Molecular Dynamics Simulations. Phys. Chem. Chem. Phys. 22, 1276–1287. 10.1039/c9cp05531b 31850422

[B59] Maier J. A. Martinez C. Kasavajhala K. Wickstrom L. Hauser K. E. Simmerling C. (2015). Ff14SB: Improving the Accuracy of Protein Side Chain and Backbone Parameters from ff99SB. J. Chem. Theory Comput. 11, 3696–3713. 10.1021/acs.jctc.5b00255 26574453 PMC4821407

[B60] Mallon M. Dutt S. Schrader T. Crowley P. B. (2016). Protein Camouflage: Supramolecular Anion Recognition by Ubiquitin. ChemBioChem 17, 774–783. 10.1002/cbic.201500477 26818656

[B61] Martos V. Bell S. C. Santos E. Isacoff E. Y. Trauner D. de Mendoza J. (2009). Calix[4]arene-based Conical-Shaped Ligands for Voltage-dependent Potassium Channels. Proc. Natl. Acad. Sci. U.S.A. 106, 10482–10486. 10.1073/pnas.0813396106 19435843 PMC2705557

[B62] McGovern R. E. Fernandes H. Khan A. R. Power N. P. Crowley P. B. (2012). Protein Camouflage in Cytochrome C-Calixarene Complexes. Nat. Chem. 4, 527–533. 10.1038/nchem.1342 22717436

[B63] McGovern R. E. Snarr B. D. Lyons J. A. McFarlane J. Whiting A. L. Paci I. (2015). Structural Study of a Small Molecule Receptor Bound to Dimethyllysine in Lysozyme. Chem. Sci. 6, 442–449. 10.1039/C4SC02383H 25530835 PMC4266562

[B64] Meiners A. Bäcker S. Hadrović I. Heid C. Beuck C. Ruiz-Blanco Y. B. (2021). Specific Inhibition of the Survivin-Crm1 Interaction by Peptide-Modified Molecular Tweezers. Nat. Commun. 12, 1505. 10.1038/s41467-021-21753-9 33686072 PMC7940618

[B65] Mikulskis P. Genheden S. Rydberg P. Sandberg L. Olsen L. Ryde U. (2012). Binding Affinities in the SAMPL3 Trypsin and Host-Guest Blind Tests Estimated with the MM/PBSA and Lie Methods. J. Comput. Aided Mol. Des. 26, 527–541. 10.1007/s10822-011-9524-z 22198518

[B66] Milroy L.-G. Grossmann T. N. Hennig S. Brunsveld L. Ottmann C. (2014). Modulators of Protein-Protein Interactions. Chem. Rev. 114, 4695–4748. 10.1021/cr400698c 24735440

[B67] Mittal S. Bravo-Rodriguez K. Sanchez-Garcia E. (2018). Mechanism of Inhibition of Beta Amyloid Toxicity by Supramolecular Tweezers. J. Phys. Chem. B 122, 4196–4205. 10.1021/acs.jpcb.7b10530 29630377

[B68] Molzan M. Schumacher B. Ottmann C. Baljuls A. Polzien L. Weyand M. (2010). Impaired Binding of 14-3-3 to C-Raf in Noonan Syndrome Suggests New Approaches in Diseases with Increased Ras Signaling. Mol. Cell. Biol. 30, 4698–4711. 10.1128/MCB.01636-09 20679480 PMC2950525

[B69] Navarrete M. Zhou Y. (2022). The 14-3-3 Protein Family and Schizophrenia. Front. Mol. Neurosci. 15, 857495. 10.3389/fnmol.2022.857495 35359567 PMC8964262

[B70] Neves J. F. Petrvalská O. Bosica F. Cantrelle F. X. Merzougui H. O'Mahony G. (2021). Phosphorylated Full‐length Tau Interacts with 14‐3‐3 Proteins via Two Short Phosphorylated Sequences, Each Occupying a Binding Groove of 14‐3‐3 Dimer. Febs J. 288, 1918–1934. 10.1111/febs.15574 32979285

[B71] Pan W. Mao L. Shi M. Fu Y. Jiang X. Feng W. (2018). The Cytochrome C-Cyclo[6]aramide Complex as a Supramolecular Catalyst in Methanol. New J. Chem. 42, 3857–3866. 10.1039/c7nj02741a

[B72] Pang Z. Sokolov M. Kubař T. Elstner M. (2022). Unravelling the Mechanism of Glucose Binding in a Protein-Based Fluorescence Probe: Molecular Dynamics Simulation with a Tailor-Made Charge Model. Phys. Chem. Chem. Phys. 24, 2441–2453. 10.1039/d1cp03733a 35019922

[B73] Park E. Rawson S. Li K. Kim B.-W. Ficarro S. B. Pino G. G.-D. (2019). Architecture of Autoinhibited and Active Braf-Mek1-14-3-3 Complexes. Nature 575, 545–550. 10.1038/s41586-019-1660-y 31581174 PMC7014971

[B74] Ravindranathan K. Tirado-Rives J. Jorgensen W. L. Guimarães C. R. W. (2011). Improving MM-GB/SA Scoring through the Application of the Variable Dielectric Model. J. Chem. Theory Comput. 7, 3859–3865. 10.1021/ct200565u 22606071 PMC3351111

[B75] Reddy A. S. Zhang S. (2013). Polypharmacology: Drug Discovery for the Future. Expert Rev. Clin. Pharmacol. 6, 41–47. 10.1586/ecp.12.74 23272792 PMC3809828

[B76] Schrader T. Bitan G. Klärner F.-G. (2016). Molecular Tweezers for Lysine and Arginine - Powerful Inhibitors of Pathologic Protein Aggregation. Chem. Commun. 52, 11318–11334. 10.1039/c6cc04640a PMC5026632 27546596

[B77] Schumacher B. Skwarczynska M. Rose R. Ottmann C. (2010). Structure of a 14-3-3σ-YAP Phosphopeptide Complex at 1.15 Å Resolution. Acta Cryst. Sect. F. Struct. Biol. Cryst. Commun. 66, 978–984. 10.1107/S1744309110025479 PMC2935210 20823509

[B78] Shi M. Xu D. (2019). Molecular Dynamics Investigations Suggest a Non-specific Recognition Strategy of 14-3-3σ Protein by Tweezer: Implication for the Inhibition Mechanism. Front. Chem. 7, 237. 10.3389/fchem.2019.00237 31058132 PMC6478809

[B79] Sonntag T. Vaughan J. M. Montminy M. (2018). 14‐3‐3 Proteins Mediate Inhibitory Effects ofcAMPon Salt‐inducible Kinases (SIKs). FEBS J. 285, 467–480. 10.1111/febs.14351 29211348 PMC5799007

[B80] Stevers L. M. Sijbesma E. Botta M. MacKintosh C. Obsil T. Landrieu I. (2018). Modulators of 14-3-3 Protein-Protein Interactions. J. Med. Chem. 61, 3755–3778. 10.1021/acs.jmedchem.7b00574 28968506 PMC5949722

[B81] Sugita Y. Okamoto Y. (1999). Replica-exchange Molecular Dynamics Method for Protein Folding. Chem. Phys. Lett. 314, 141–151. 10.1016/S0009-2614(99)01123-9

[B82] Supuran C. T. (2020). Exploring the Multiple Binding Modes of Inhibitors to Carbonic Anhydrases for Novel Drug Discovery. Expert Opin. Drug Discov. 15, 671–686. 10.1080/17460441.2020.1743676 32208982

[B83] Talbiersky P. Bastkowski F. Klärner F.-G. Schrader T. (2008). Molecular Clip and Tweezer Introduce New Mechanisms of Enzyme Inhibition. J. Am. Chem. Soc. 130, 9824–9828. 10.1021/ja801441j 18605724

[B84] Tiwary P. Limongelli V. Salvalaglio M. Parrinello M. (2015). Kinetics of Protein-Ligand Unbinding: Predicting Pathways, Rates, and Rate-Limiting Steps. Proc. Natl. Acad. Sci. U.S.A. 112, E386–E391. 10.1073/pnas.1424461112 25605901 PMC4321287

[B85] Torrie G. M. Valleau J. P. (1977). Nonphysical Sampling Distributions in Monte Carlo Free-Energy Estimation: Umbrella Sampling. J. Comput. Phys. 23, 187–199. 10.1016/0021-9991(77)90121-8

[B86] Trusch F. Kowski K. Bravo-Rodriguez K. Beuck C. Sowislok A. Wettig B. (2016). Molecular Tweezers Target a Protein-Protein Interface and Thereby Modulate Complex Formation. Chem. Commun. 52, 14141–14144. 10.1039/c6cc08039a 27869276

[B87] Urbach A. R. Ramalingam V. (2011). Molecular Recognition of Amino Acids, Peptides, and Proteins by Cucurbit[n]uril Receptors. Isr. J. Chem. 51, 664–678. 10.1002/ijch.201100035

[B88] van Dun S. Ottmann C. Milroy L.-G. Brunsveld L. (2017). Supramolecular Chemistry Targeting Proteins. J. Am. Chem. Soc. 139, 13960–13968. 10.1021/jacs.7b01979 28926241 PMC5639466

[B89] Verdoodt B. Benzinger A. Popowicz G. M. Holak T. A. Hermeking H. (2006). Characterization of 14-3-3sigma Dimerization Determinants: Requirement of Homodimerization for Inhibition of Cell Proliferation. Cell Cycle 5, 2920–2926. 10.4161/cc.5.24.3571 17172876

[B90] Vöpel T. Bravo-Rodriguez K. Mittal S. Vachharajani S. Gnutt D. Sharma A. (2017). Inhibition of Huntingtin Exon-1 Aggregation by the Molecular Tweezer CLR01. J. Am. Chem. Soc. 139, 5640–5643. 10.1021/jacs.6b11039 28406616 PMC5506490

[B91] Wakchaure P. D. Ganguly B. (2022). Deciphering the Mechanism of Action of 5fdqd and the Design of New Neutral Analogues for the Fmn Riboswitch: a Well-Tempered Metadynamics Simulation Study. Phys. Chem. Chem. Phys. 24, 817–828. 10.1039/d1cp01348c 34928280

[B92] Wang J. Wang W. Kollman P. A. Case D. A. (2006). Automatic Atom Type and Bond Type Perception in Molecular Mechanical Calculations. J. Mol. Graph. Model. 25, 247–260. 10.1016/j.jmgm.2005.12.005 16458552

[B93] Wang J. Wolf R. M. Caldwell J. W. Kollman P. A. Case D. A. (2004). Development and Testing of a General Amber Force Field. J. Comput. Chem. 25, 1157–1174. 10.1002/jcc.20035 15116359

[B94] Wang Q. An X. Xu J. Wang Y. Liu L. Leung E. L.-H. (2018). Classical Molecular Dynamics and Metadynamics Simulations Decipher the Mechanism of CBP30 Selectively Inhibiting CBP/p300 Bromodomains. Org. Biomol. Chem. 16, 6521–6530. 10.1039/c8ob01526k 30160288

[B95] Wang R. Xu D. (2019). Molecular Dynamics Investigations of Oligosaccharides Recognized by Family 16 and 22 Carbohydrate Binding Modules. Phys. Chem. Chem. Phys. 21, 21485–21496. 10.1039/c9cp04673a 31535114

[B96] Waterhouse A. Bertoni M. Bienert S. Studer G. Tauriello G. Gumienny R. (2018). SWISS-MODEL: Homology Modelling of Protein Structures and Complexes. Nucleic Acids Res. 46, W296–W303. 10.1093/nar/gky427 29788355 PMC6030848

[B97] Weil T. Gross R. Röcker A. Bravo-Rodriguez K. Heid C. Sowislok A. (2020). Supramolecular Mechanism of Viral Envelope Disruption by Molecular Tweezers. J. Am. Chem. Soc. 142, 17024–17038. 10.1021/jacs.0c06400 32926779 PMC7523239

[B98] Wilch C. Talbiersky P. Berchner‐Pfannschmidt U. Schaller T. Kirsch M. Klärner F. G. (2017). Molecular Tweezers Inhibit PARP‐1 by a New Mechanism. Eur. J. Org. Chem. 2017, 2223–2229. 10.1002/ejoc.201601596

[B99] Wilker E. W. Grant R. A. Artim S. C. Yaffe M. B. (2005). A Structural Basis for 14-3-3σ Functional Specificity. J. Biol. Chem. 280, 18891–18898. 10.1074/jbc.M500982200 15731107

[B100] Williams G. T. Haynes C. J. E. Fares M. Caltagirone C. Hiscock J. R. Gale P. A. (2021). Advances in Applied Supramolecular Technologies. Chem. Soc. Rev. 50, 2737–2763. 10.1039/d0cs00948b 33438685

[B101] Winter M. Rokavec M. Hermeking H. (2021). 14-3-3σ Functions as an Intestinal Tumor Suppressor. Cancer Res. 81, 3621–3634. 10.1158/0008-5472.Can-20-4192 34224368

[B102] Yang H. Yuan B. Zhang X. Scherman O. A. (2014). Supramolecular Chemistry at Interfaces: Host-Guest Interactions for Fabricating Multifunctional Biointerfaces. Acc. Chem. Res. 47, 2106–2115. 10.1021/ar500105t 24766328

[B103] Yang H. Zhao R. Lee M.-H. (2006a). 14-3-3σ, a P53 Regulator, Suppresses Tumor Growth of Nasopharyngeal Carcinoma. Mol. Cancer Ther. 5, 253–260. 10.1158/1535-7163.MCT-05-0395 16505098

[B104] Yang X. Lee W. H. Sobott F. Papagrigoriou E. Robinson C. V. Grossmann J. G. (2006b). Structural Basis for Protein-Protein Interactions in the 14-3-3 Protein Family. Proc. Natl. Acad. Sci. U.S.A. 103, 17237–17242. 10.1073/pnas.0605779103 17085597 PMC1859916

[B105] Zhang Y. Cao Z. Zhang J. Z. Xia F. (2020). Double-well Ultra-coarse-grained Model to Describe Protein Conformational Transitions. J. Chem. Theory Comput. 16, 6678–6689. 10.1021/acs.jctc.0c00551 32926616

[B106] Zhao J. Du Y. Horton J. R. Upadhyay A. K. Lou B. Bai Y. (2011). Discovery and Structural Characterization of a Small Molecule 14-3-3 Protein-Protein Interaction Inhibitor. Proc. Natl. Acad. Sci. U.S.A. 108, 16212–16216. 10.1073/pnas.1100012108 21908710 PMC3182712

[B107] Zhu K. Shirts M. R. Friesner R. A. (2007). Improved Methods for Side Chain and Loop Predictions via the Protein Local Optimization Program: Variable Dielectric Model for Implicitly Improving the Treatment of Polarization Effects. J. Chem. Theory Comput. 3, 2108–2119. 10.1021/ct700166f 26636204

