# Supplementary material for: Exploring the Binding Mechanism of a Supramolecular Tweezer CLR01 to 14-3-3σ Protein via Well-Tempered Metadynamics
Source: Front Chem. 2022 May 12;10:921695. doi: 10.3389/fchem.2022.921695 (PMC9133541; doi:10.3389/fchem.2022.921695)
Supplement: Supplementary file 1 [file DataSheet1.pdf]

Supporting Information

Exploring the Binding Mechanism of a Supramolecular Tweezer CLR01 to 14-3-3σ Protein via Well-tempered Metadynamics

Xin Zhou[1], Mingsong Shi[2], Xin Wang[1*] and Dingguo Xu[1,3*]

[1] College of Chemistry, MOE Key Laboratory of Green Chemistry and Technology, Sichuan University, Chengdu, Sichuan, 610064, China

[2]State Key Laboratory of Biotherapy/Collaborative Innovation Center of Biotherapy and Cancer Center, West China Hospital, Sichuan University, Chengdu, Sichuan, 610041, China

[3]Research Center for Material Genome Engineering, Sichuan University, Chengdu, Sichuan, 610065, China

*To whom correspondence should be addressed:
wangxin@scu.edu.cn (X. W.); dgxu@scu.edu.cn (D. X.)

Contents

Figure S1. Crystal structures of tweezer/14-3-3. ....S4
Figure S2. Superimposed structures of 14-3-3ζ from published crystal structures (PDB: 5M37) and 14-3-3σ from published crystal structures (PDB: 1YZ5)..........................S5
Figure S3. Distributions of 17 lysine residues on 14-3-3σ. S6
Figure S4. Initial structures of tweezer with 14-3-3σ. S7
Figure S5. Collective variables selected for metadynamics in this work. S8
Figure S6. Two-dimensional free energy landscapes for the tweezer to these 13 lysine sites. S10
Figure S7. Convergence plot of the binding free energy (ΔG_b) at different time for tweezer binding to (A) K32, (B) K68, (C) K77, (D) K87, (E) K141, (F) K159, (G) K160, (H) K214. ....S11
Figure S8. Evolution of distance (CV1) and coordination number (CV2) for tweezer binding to lysine sites along simulation time..S14
Figure S9. Superimposed structures of the unbound state, transition state and bound state of tweezer binding to the strong binding sites (A) K214, (B) K77, (C) K141, and (D) K160. ....S15
Figure S10. Structure characteristics of K77 in apo-14-3-3σ. S16
Figure S11. Other representative snapshots for tweezer/K160 system on 14-3-3σ...S17
Figure S12. Superimposed structures of the unbound state, transition state and bound state of tweezer binding to (A) K32, (B) K68, and unbound state, transition state, semi-bound state and bound state of tweezer binding to (C) K87, and (D) K159.............S18
Figure S13. Semi-bound state for tweezer binding to (A) K87, (B)K159. ....S19
Figure S14. Evolution of the distance (CV1) when tweezer bound to (A) K87 and (B) K159 along the simulation time, respectively...S20
Figure S15. Steric hindrance for (A) K9, (B) K122, (C) K124, and (D) K140. ....S21
Figure S16. Representative snapshots of the tweezer binding to (A) K11, (B) K27, (C) K49, (D) K109, (E) K195 along the simulation time. S22
Figure S17. Electrostatic and hydrophobic interactions between tweezer and other amino acids for K109 site. S23
Figure S18. Steric hindrance and electrostatic attraction near the K195 (A) in the apo-14-3-3σ and (B) in the semi-bound state. S24
Figure S19. Steric hindrance for K9, K11, K27, K49, K122, K124, K140, and K195 on 14-3-3σ...S25

Figure S20. Hydrogen bonds for the additional residues..S26
Figure S21. Sites of the lysine residues on 14-3-3σ with the active regions. S27
Figure S22. Superimposed structures of MT-ExoS, CLR01, ExoS binding to 14-3-3. ....S28
Table S1. Simulation time of tweezer binding to lysine residues on 14-3-3σ for metadynamics. S29
Table S2. Binding free energy (ΔG) and binding energy barrier for tweezer binding to lysine residues from metadynamics. S30
Table S3. Distance of the upward phosphate group (P1) of tweezer and the ammonium group of lysine residue in the bound state. S31

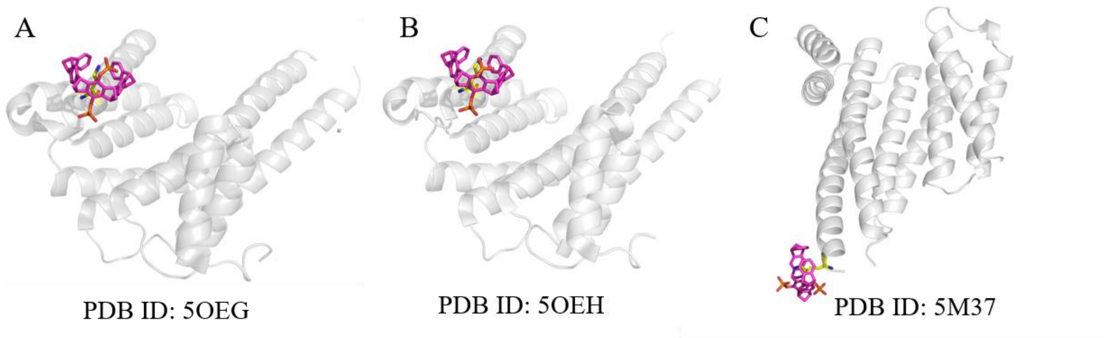

Figure S1. Crystal structures of tweezer/14-3-3.

(A) Structure of tweezer/K214 (14-3-3σ) from published crystal structures (PDB ID of 5OEG with a resolution of 3.15 Å). (B) Structure of tweezer/K214 (14-3-3σ) from published crystal structures (PDB ID of 5OEH with a resolution of 2.35 Å). (C) Structure of tweezer/K74 (14-3-3ζ) from published crystal structures (PDB ID of 5M37 with a resolution of 2.35 Å). The tweezer molecule and the binding lysine residue are plotted using stick style, in which tweezer is colored in magentas, and the lysine residue is colored in yellow.

PDB ID: 5OEG, Bier, D.; Rose, R.; Bravo-Rodriguez, K.; Bartel, M.; Ramirez-Anguita, J. M.; Dutt, S.; Wilch, C.; Klarner, F. G.; Sanchez-Garcia, E.; Schrader, T.; al., e., Molecular tweezers modulate 14-3-3 protein-protein interactions. Nat. Chem. 2013, 5, 234–239.

PDB ID: 5OEH, Bier, D.; Rose, R.; Bravo-Rodriguez, K.; Bartel, M.; Ramirez-Anguita, J. M.; Dutt, S.; Wilch, C.; Klarner, F. G.; Sanchez-Garcia, E.; Schrader, T.; al., e., Molecular tweezers modulate 14-3-3 protein-protein interactions. Nat. Chem. 2013, 5, 234–239.

PDB ID: 5M37, Bier, D.; Mittal, S.; Bravo-Rodriguez, K.; Sowislok, A.; Guillory, X.; Briels, J.; Heid, C.; Bartel, M.; Wettig, B.; Brunsveld, L.; al., e., The molecular tweezer CLR01 stabilizes a disordered protein−protein interface. J. Am. Chem. Soc. 2017, 139, 16256−16263.

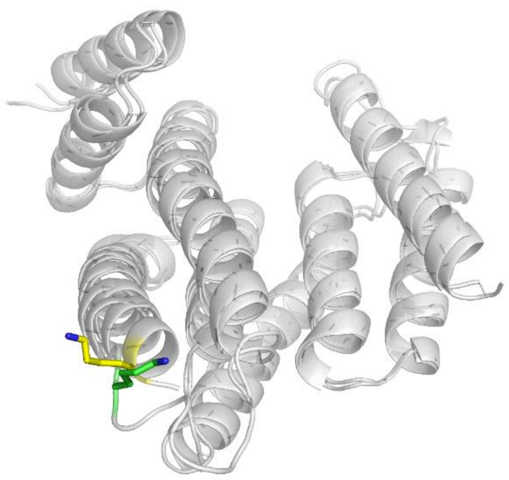

Figure S2. Superimposed structures of 14-3-3ζ from published crystal structures (PDB: 5M37) and 14-3-3σ from published crystal structures (PDB: 1YZ5).

The K74 on 14-3-3ζ and K77 on 14-3-3σ are plotted by stick style, and colored with yellow, and green, respectively.

PDB ID: 5M37, Bier, D.; Mittal, S.; Bravo-Rodriguez, K.; Sowislok, A.; Guillory, X.; Briels, J.; Heid, C.; Bartel, M.; Wettig, B.; Brunsveld, L.; al., e., The molecular tweezer CLR01 stabilizes a disordered protein−protein interface. J. Am. Chem. Soc. 2017, 139, 16256−16263.

PDB ID: 1YZ5, Benzinger, A.; Popowicz, G. M.; Joy, J. K.; Majumdar, S.; Holak, T. A.; Hermeking, H., The crystal structure of the non-liganded 14-3-3σ protein: insights into determinants of isoform specific ligand binding and dimerization. Cell Res. 2005, 15, 219–227.

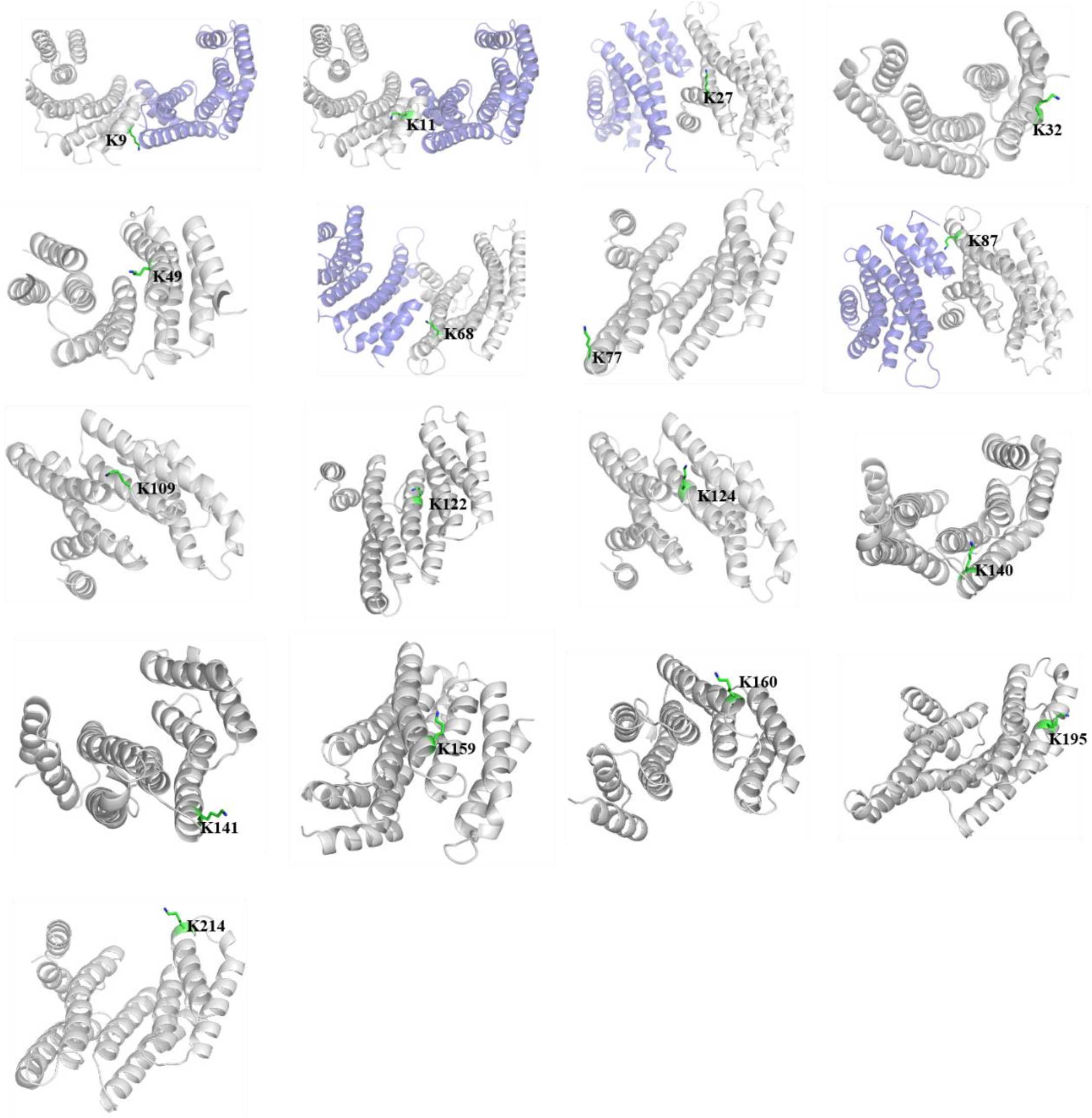

Figure S3. Distributions of 17 lysine residues on 14-3-3σ.

These lysine residues are plotted using stick style and colored in green. The A chain of 14-3-3σ is colored in gray, and the B chain of 14-3-3σ is colored in purple.

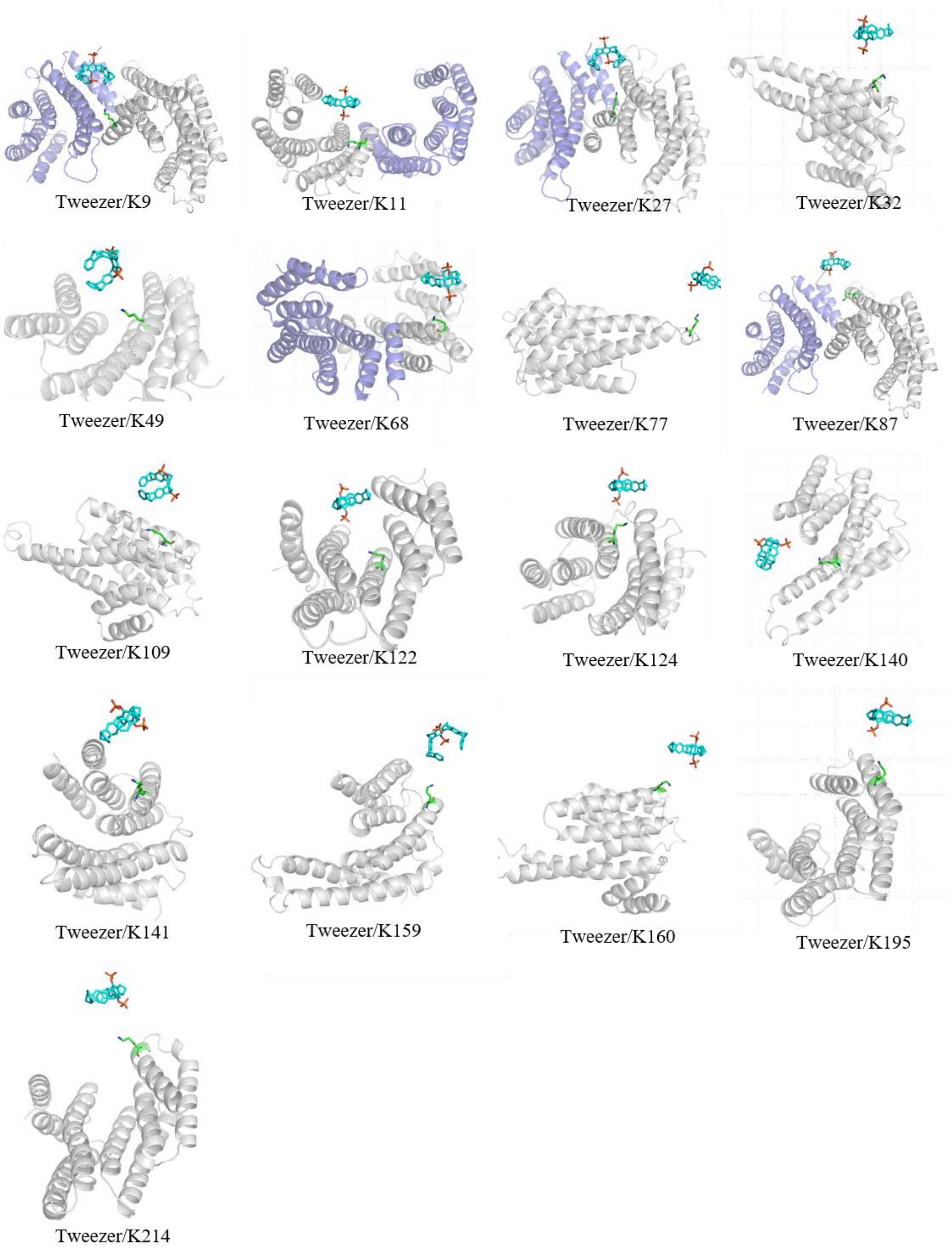

Figure S4. Initial structures of tweezer with 14-3-3σ.

The tweezer was manually move to a position more than 12 Å from the selected lysine residue through VMD 1.9.1 software.

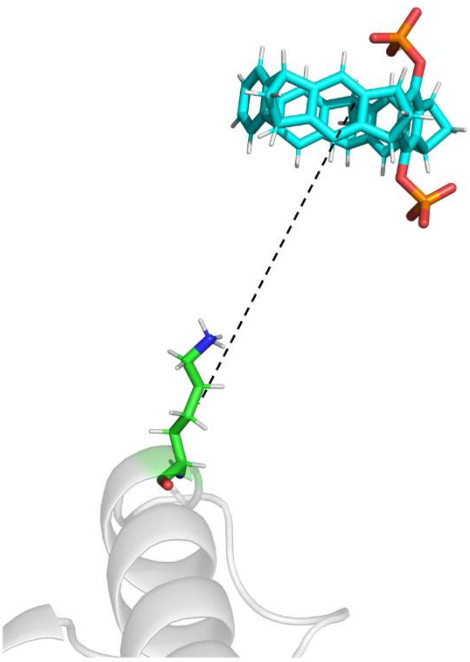

Figure S5. Collective variables selected for metadynamics in this work.

Black dotted lines represent the CV1. CV1 was the distance between the center of mass (COM) of the lysine side chain and the COM of the tweezer ring excluding the two phosphate groups. CV2 was the coordination number between the heavy atom in the side chain of lysine and the carbon atom in the tweezer ring.

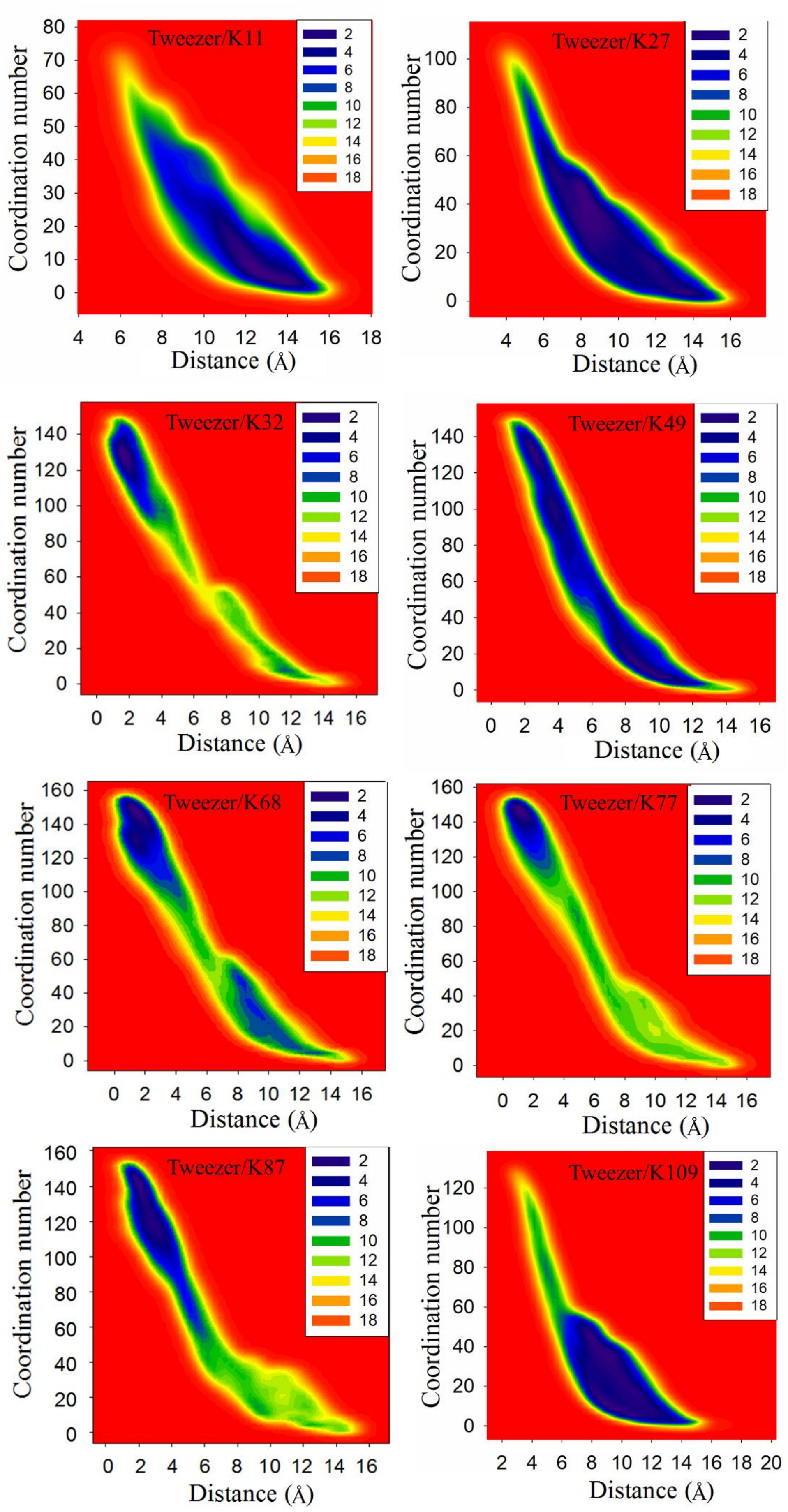
Tweezer/K11
Coordination number
Distance (Å)
80
70
60
50
40
30
20
10
0
4
6
8
10
12
14
16
18
2
4
6
8
10
12
14
16
18
Tweezer/K27
Coordination number
Distance (Å)
100
80
60
40
20
0
4
6
8
10
12
14
16
Tweezer/K32
Coordination number
Distance (Å)
140
120
100
80
60
40
20
0
0
2
4
6
8
10
12
14
16
Tweezer/K49
Coordination number
Distance (Å)
Tweezer/K68
Coordination number
Distance (Å)
160
Tweezer/K77
Coordination number
Distance (Å)
Tweezer/K87
Coordination number
Distance (Å)
Tweezer/K109
Coordination number
Distance (Å)
18
20

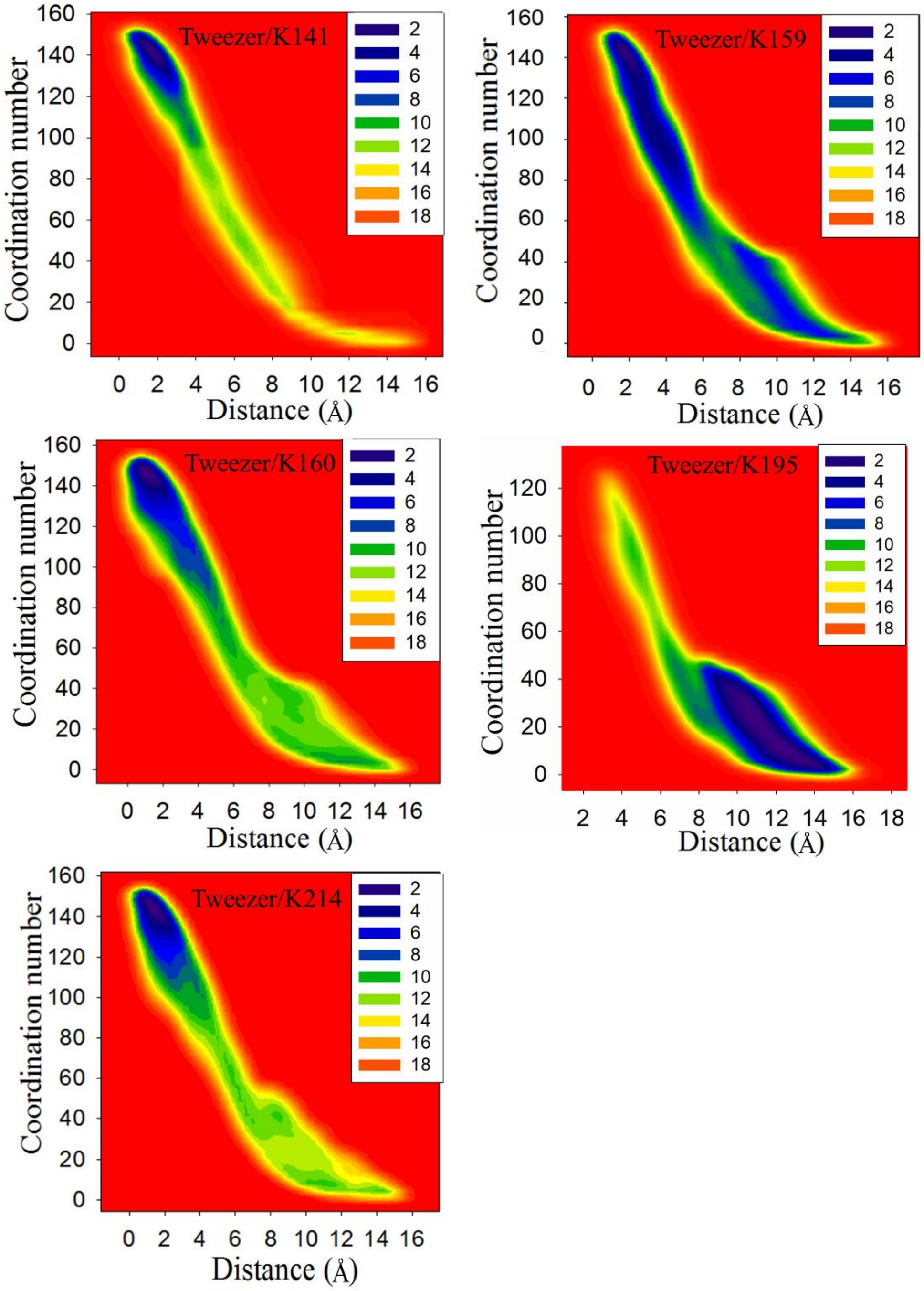

Figure S6. Two-dimensional free energy landscapes for the tweezer to these 13 lysine sites.

Free energies are in kcal/mol. The binding free energy value can be calculated from the free energy of the bound state minus the free energy of the unbound state. Herein, it can be found that K11, K27, K49, K109, K195 could not be recognized by tweezer with the binding free energies are greater than zero.

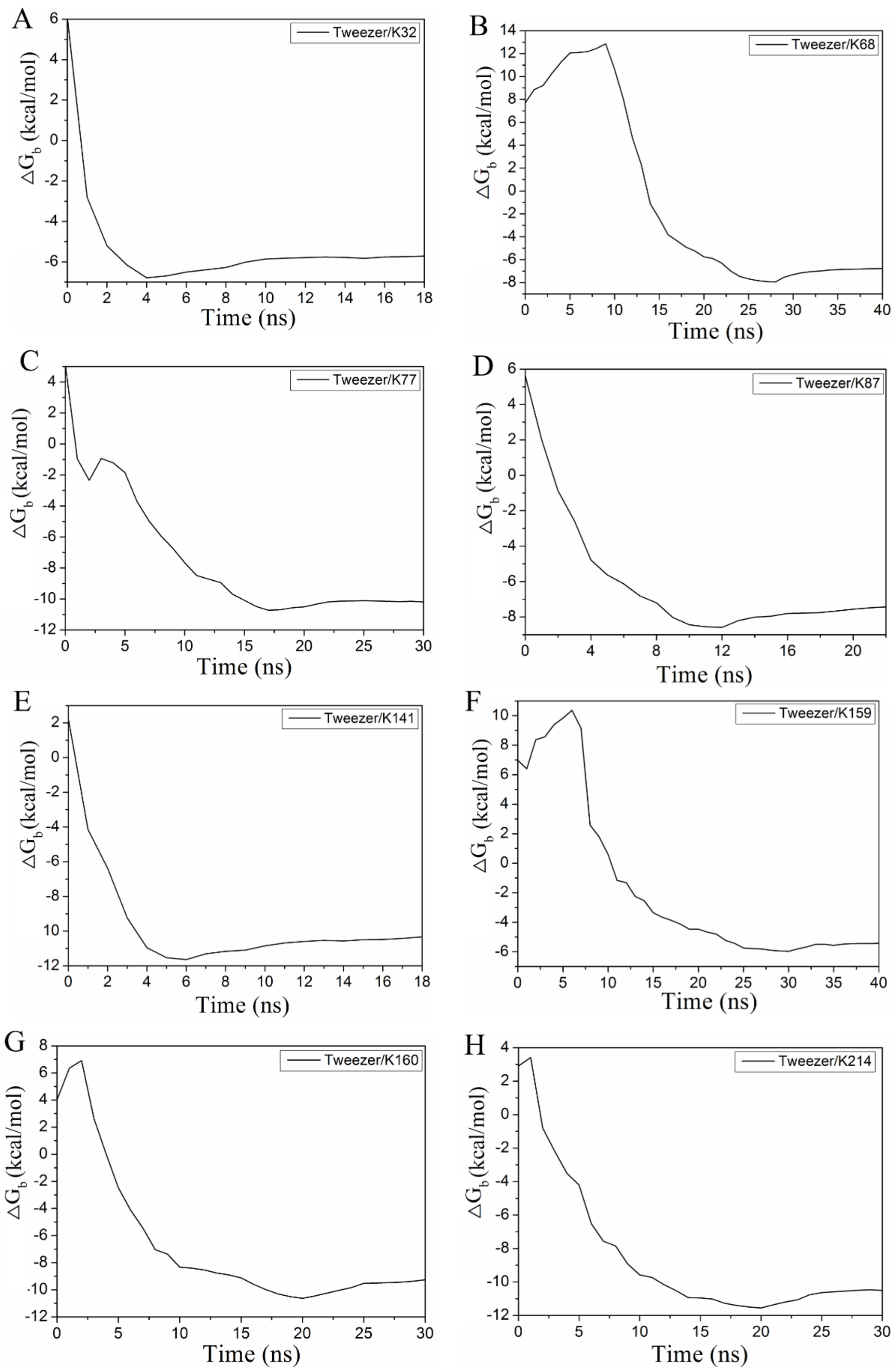

Figure S7. Convergence plot of the binding free energy (ΔG_b) at different time for tweezer binding to (A) K32, (B) K68, (C) K77, (D) K87, (E) K141, (F) K159, (G) K160, (H) K214.

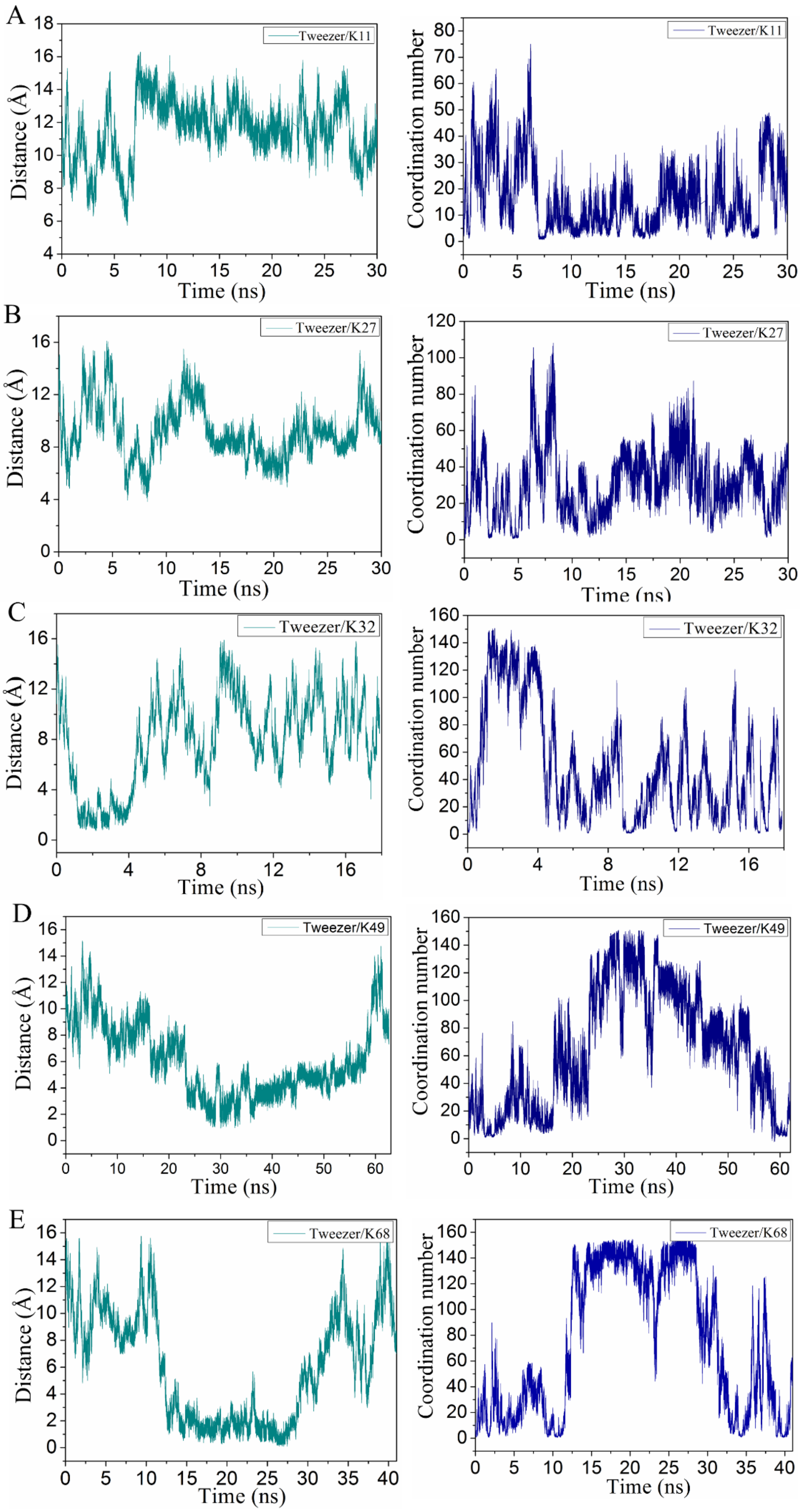
A
B
C
D
E
Distance (Å)
Coordination number
Time (ns)
Tweezer/K11
Tweezer/K27
Tweezer/K32
Tweezer/K49
Tweezer/K68

Continue

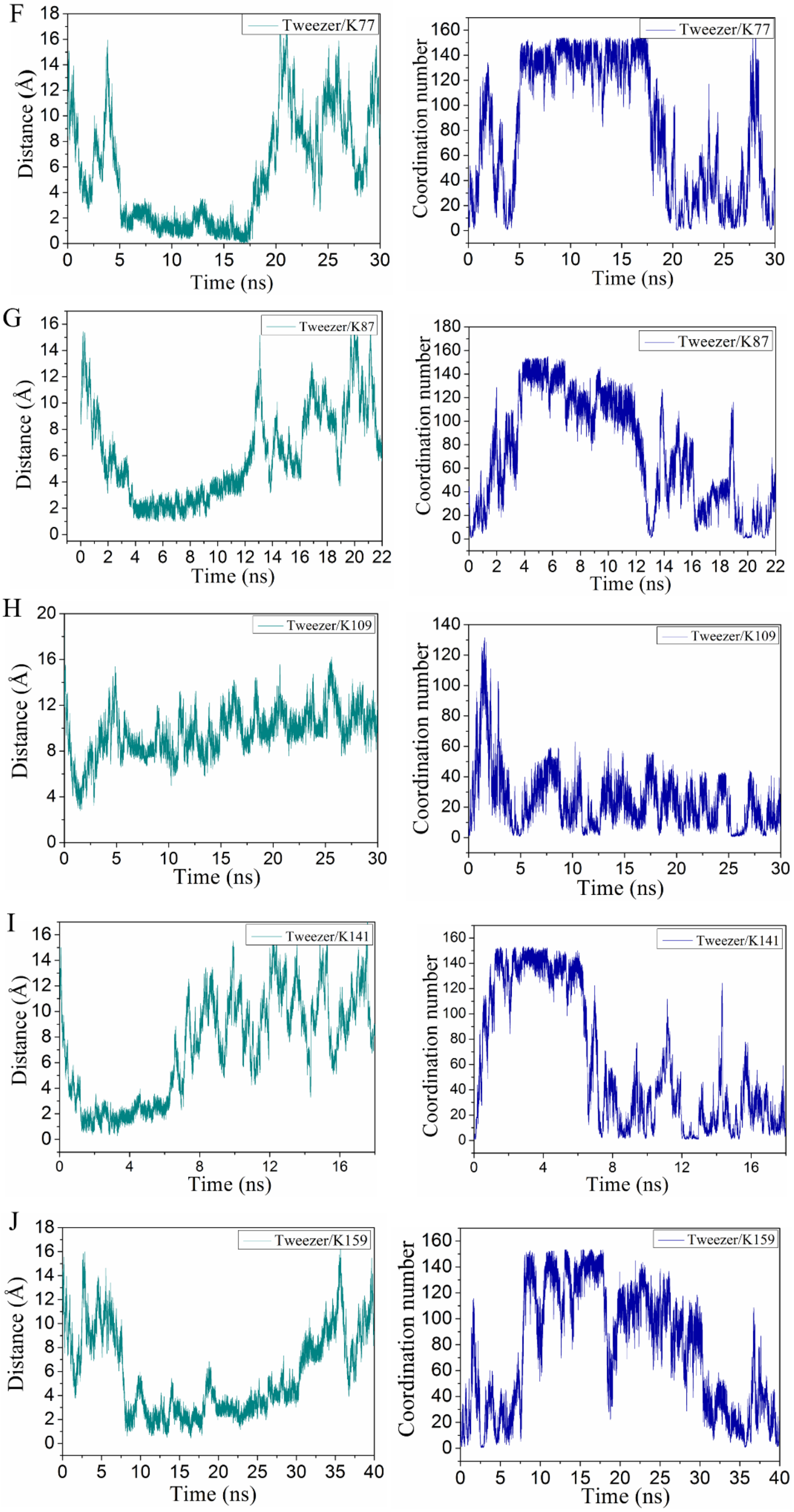
F
Distance (Å)
Time (ns)
Tweezer/K77
Coordination number
Time (ns)
Tweezer/K77
G
Distance (Å)
Time (ns)
Tweezer/K87
Coordination number
Time (ns)
Tweezer/K87
H
Distance (Å)
Time (ns)
Tweezer/K109
Coordination number
Time (ns)
Tweezer/K109
I
Distance (Å)
Time (ns)
Tweezer/K141
Coordination number
Time (ns)
Tweezer/K141
J
Distance (Å)
Time (ns)
Tweezer/K159
Coordination number
Time (ns)
Tweezer/K159

Continue

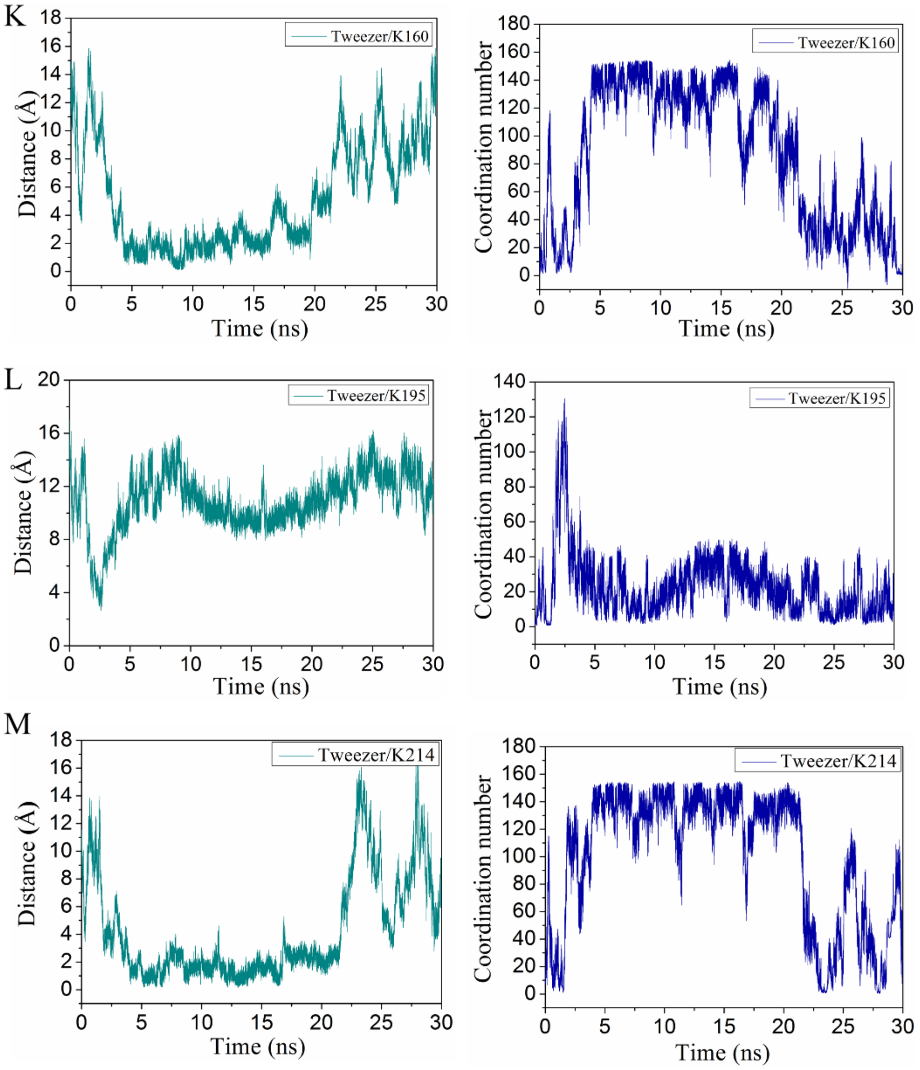

Figure S8. Evolution of distance (CV1) and coordination number (CV2) for tweezer binding to lysine sites along simulation time.

These lysine sites including (A) K11, (B) K27, (C) K32, (D) K49, (E) K68, (F) K77, (G) K87, (H) K 109, (I) K141, (J) K159, (K) K160, (L) K195, and (M) K214. It is obvious that only the eight lysine sits, including K32, K68, K77, K87, K141, K159, K160, and K214 could form stable complex with tweezer. Although K49, K109, and K195 can form very transient inclusion complexes with tweezer, they do not contribute reasonable binding free energy. In addition, due to the obvious steric hindrance for the K9, K122, K124, and K140, their association with tweezer was not considered here.

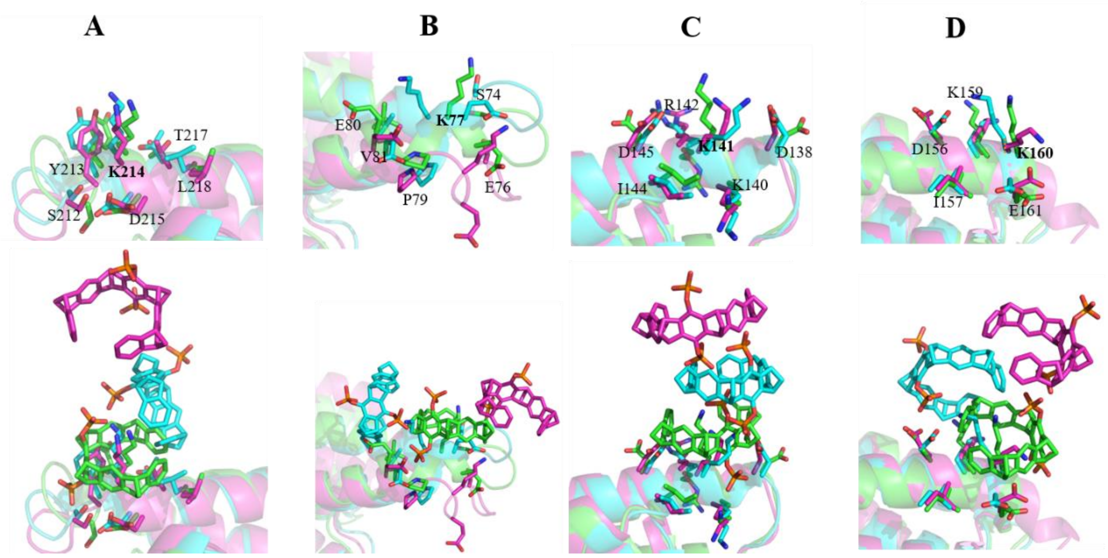

Figure S9. Superimposed structures of the unbound state, transition state and bound state of tweezer binding to the strong binding sites (A) K214, (B) K77, (C) K141, and (D) K160.

The first row mainly shows the changes of key residues in the binding process for each lysine site. The second row shows the changes of tweezer position in the binding process for each lysine site. Tweezer molecule and key residues are plotted using stick style. The unbound state, transition state and bound state are colored in magentas, blue, green, respectively.

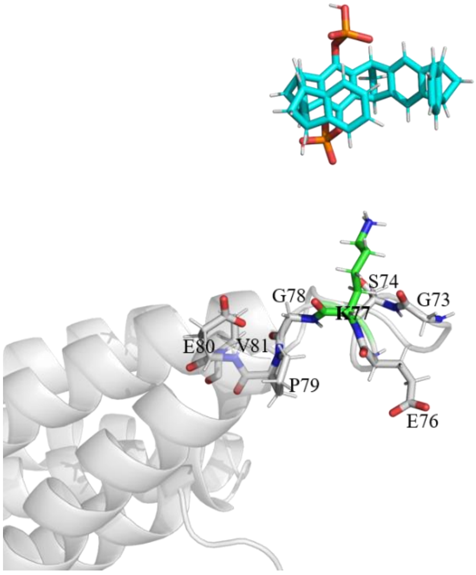

Figure S10. Structure characteristics of K77 in apo-14-3-3σ.

Around K77, there are acidic residues E76 and E80, but K77 hardly interacts with them. In addition, some short chain residues around K77, such as G73, S74, G78, and P79, further promote the complete exposure of K77 to the solvent environment.

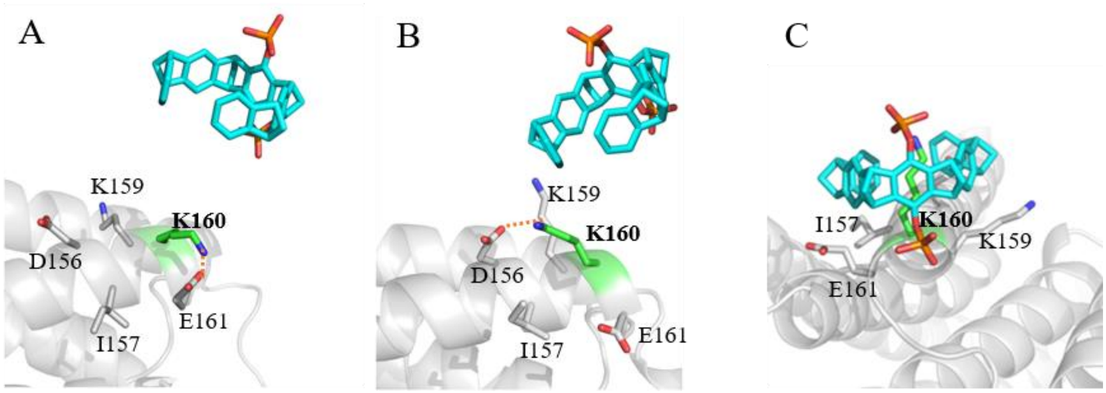

Figure S11. Other representative snapshots for tweezer/K160 system on 14-3-3σ.
(A) K160 forms hydrogen bonds with E161 before binding. (B) K160 forms hydrogen bonds with D156 before binding. (C) The downward phosphate group of tweezer is prefer to toward the solvent than forming electrostatic interaction with K159 from the mismatch of space and molecular size

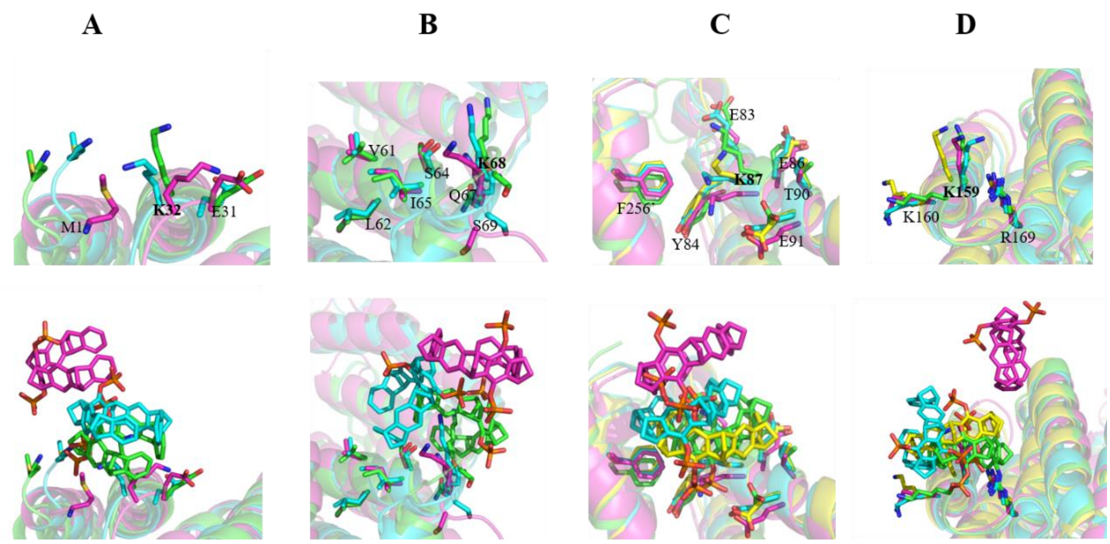

Figure S12. Superimposed structures of the unbound state, transition state and bound state of tweezer binding to (A) K32, (B) K68, and unbound state, transition state, semi-bound state and bound state of tweezer binding to (C) K87, and (D) K159.

The first row mainly shows the changes of key residues in the binding process for each site. The second row shows the changes of tweezer position in the binding process for each lysine site. The tweezer molecule and key residues are plotted using stick style. The unbound state, transition state, semi-bound state and bound state are colored in magenta, blue, yellow, and green, respectively.

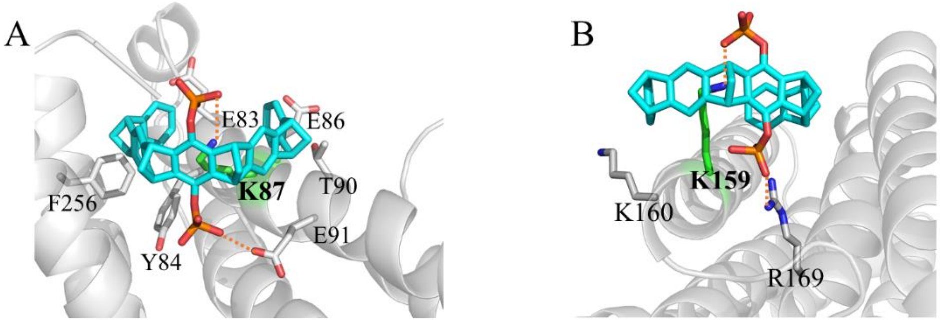

Figure S13. Semi-bound state for tweezer binding to (A) K87, (B)K159.
The key residues and tweezer are plotted using stick style. K87 and K159 are colored in green, the other residues and tweezer are colored in grey and blue, respectively.

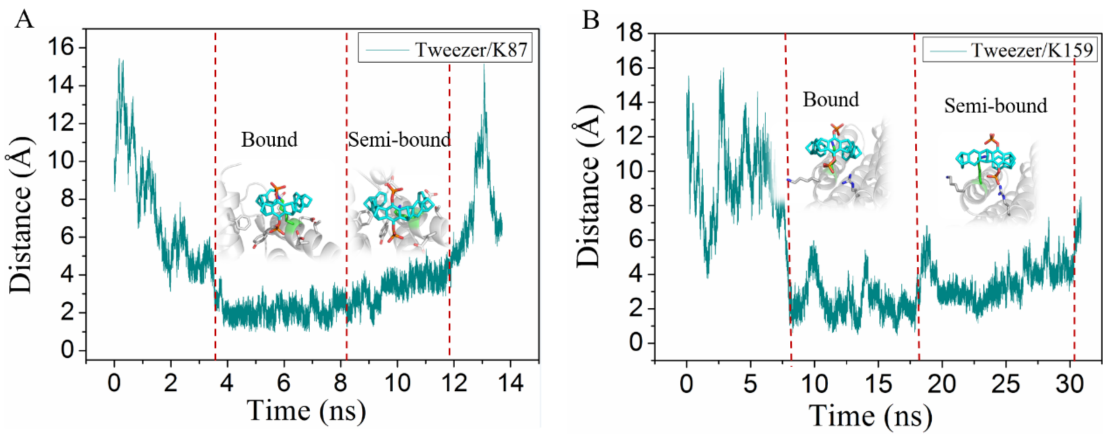

Figure S14. Evolution of the distance (CV1) when tweezer bound to (A) K87 and (B) K159 along the simulation time, respectively.

The bound state and semi-bound state are classed out according to the simulation time.

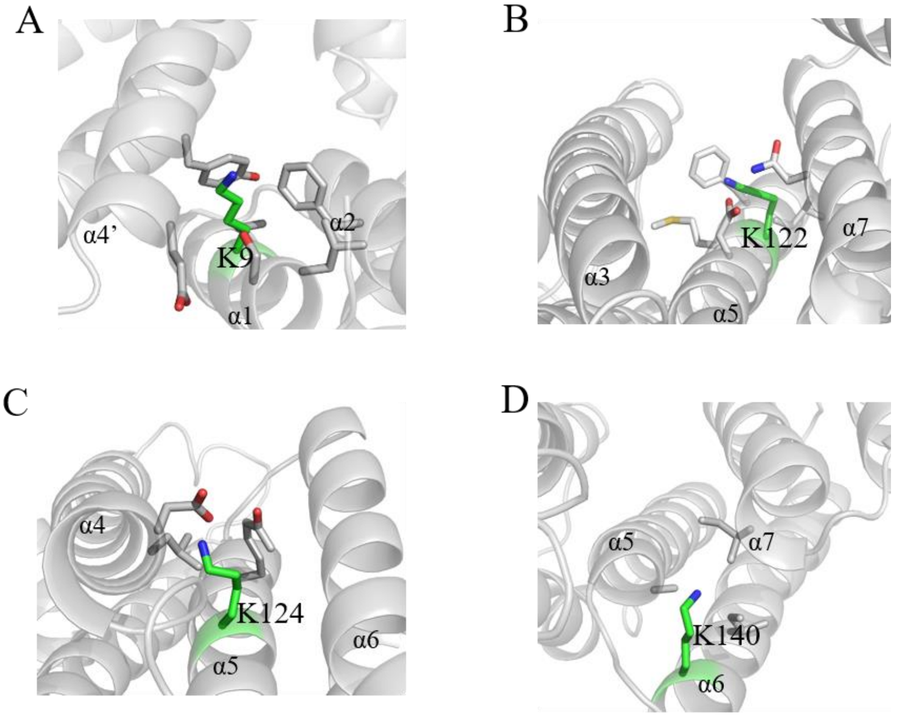

Figure S15. Steric hindrance for (A) K9, (B) K122, (C) K124, and (D) K140. α1-α7 represent the helix on the A chain of 14-3-3σ, and α4’ represents the helix on the B chain of 14-3-3σ.

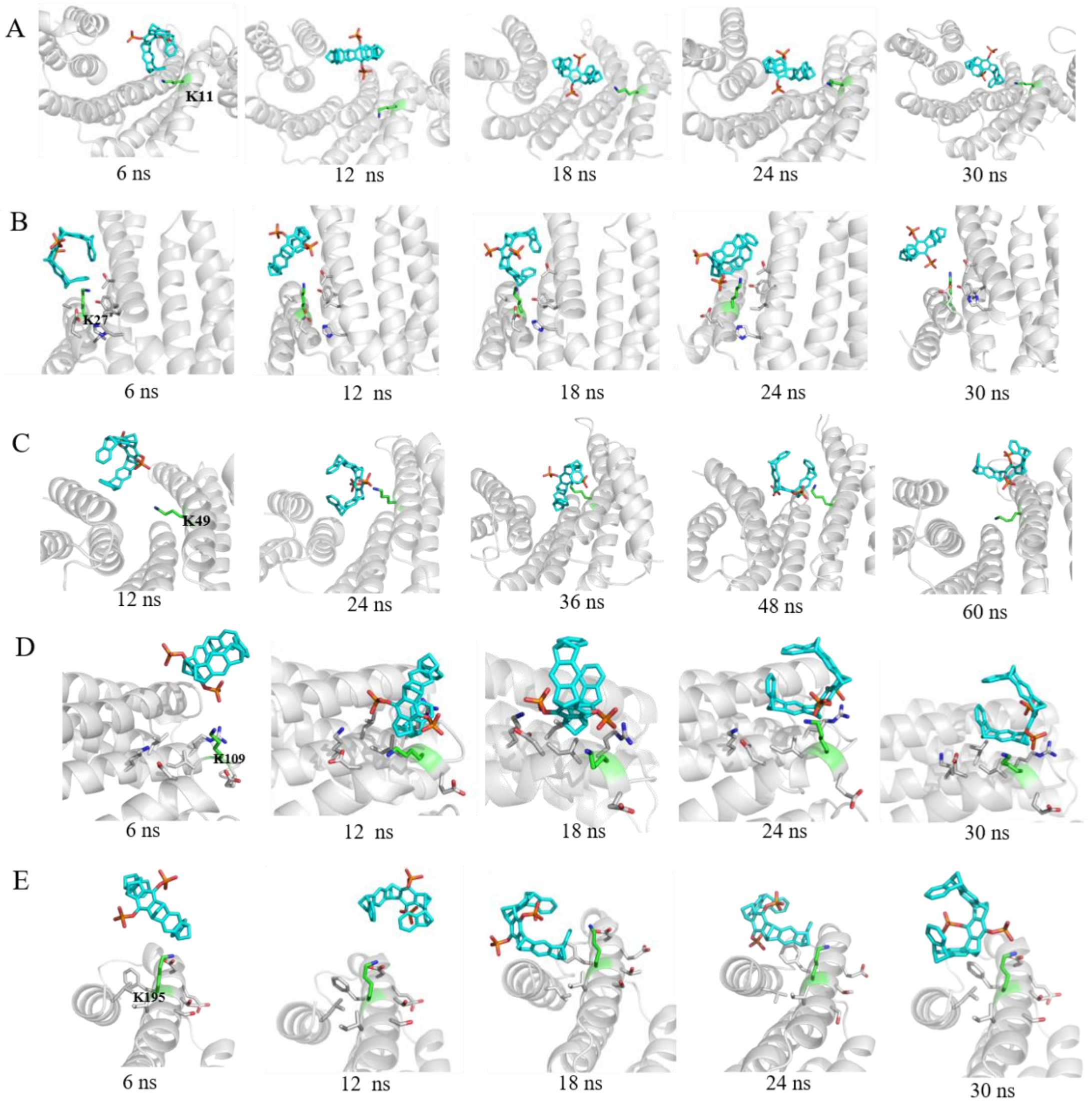

Figure S16. Representative snapshots of the tweezer binding to (A) K11, (B) K27, (C) K49, (D) K109, (E) K195 along the simulation time.

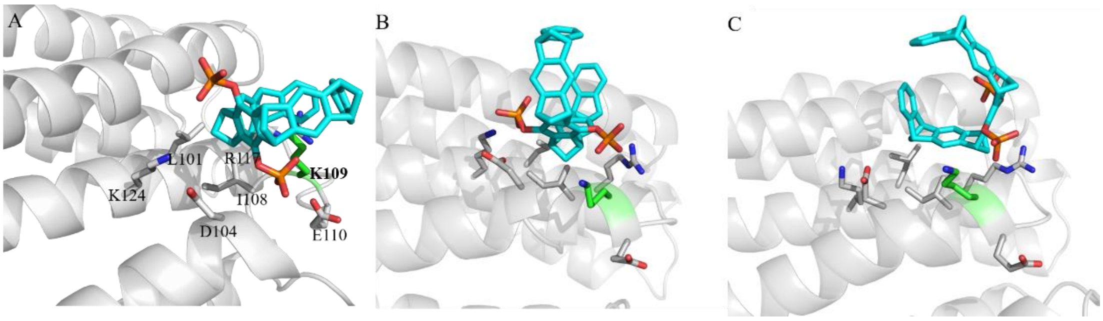

Figure S17. Electrostatic and hydrophobic interactions between tweezer and other amino acids for K109 site.

(A) Repulsion between E110 and the phosphate group of the tweezer.

(B-C) Hydrophobic environment and electrostatic attraction near the K109.

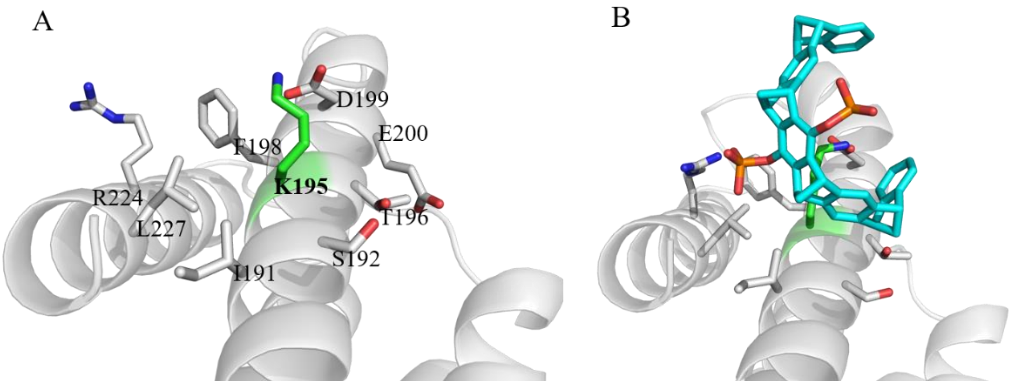

Figure S18. Steric hindrance and electrostatic attraction near the K195 (A) in the apo-14-3-3σ and (B) in the semi-bound state.

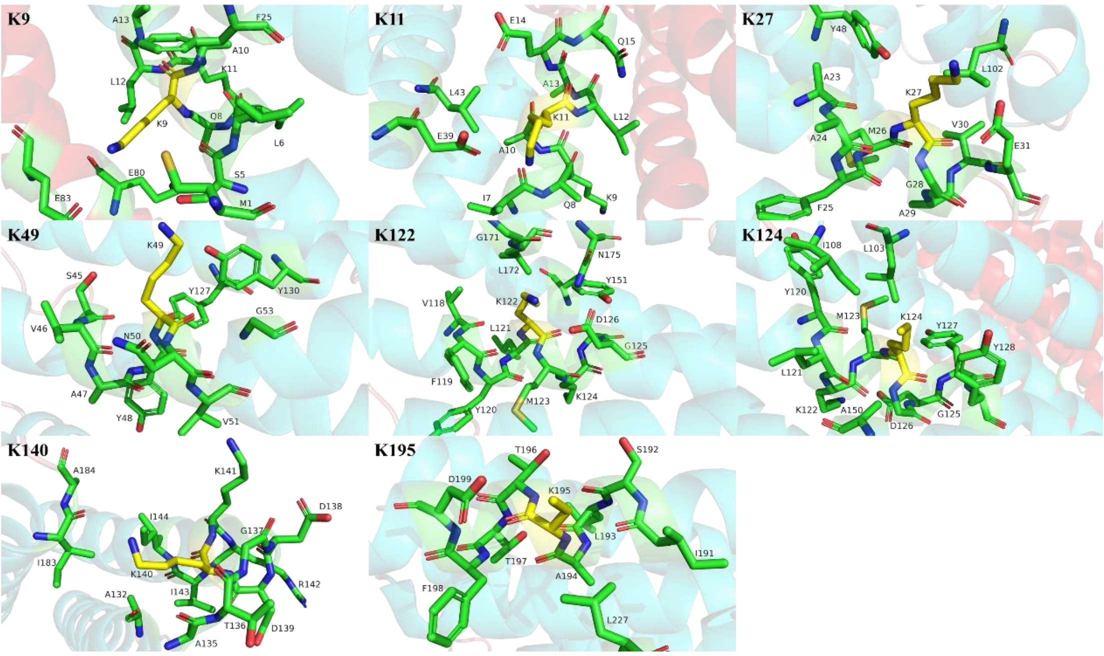

Figure S19. Steric hindrance for K9, K11, K27, K49, K122, K124, K140, and K195 on 14-3-3σ.

The lysine residue shows with stick style and yellow color. The residues around 4 Å of the lysine are shown stick style and green color. The chain A of 14-3-3σ shows with cyan color and chain B with red color. The dimer 14-3-3σ was obtained with 1YZ5 (PDB ID).

1YZ5: Benzinger, A.; Popowicz, G. M.; Joy, J. K.; Majumdar, S.; Holak, T. A.; Hermeking, H., The crystal structure of the non-liganded 14-3-3σ protein: insights into determinants of isoform specific ligand binding and dimerization. Cell Res. 2005, 15, 219–227.

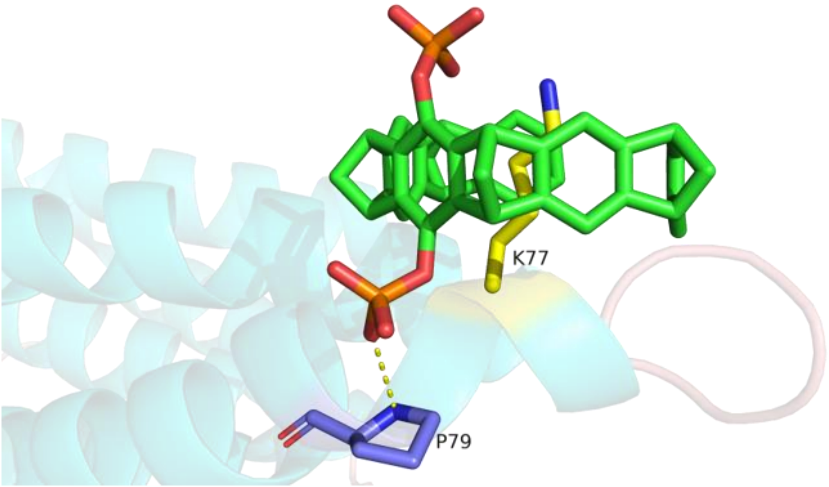

Figure S20. Hydrogen bonds for the additional residues.

The lysine residue shows with stick style and yellow color. The tweezer is shown stick style and green color. The 14-3-3σ shows with cyan color. The additional residues with hydrogen bonds are shown with stick and blue. The tweezer/14-3-3σ was obtained from the metadynamics.

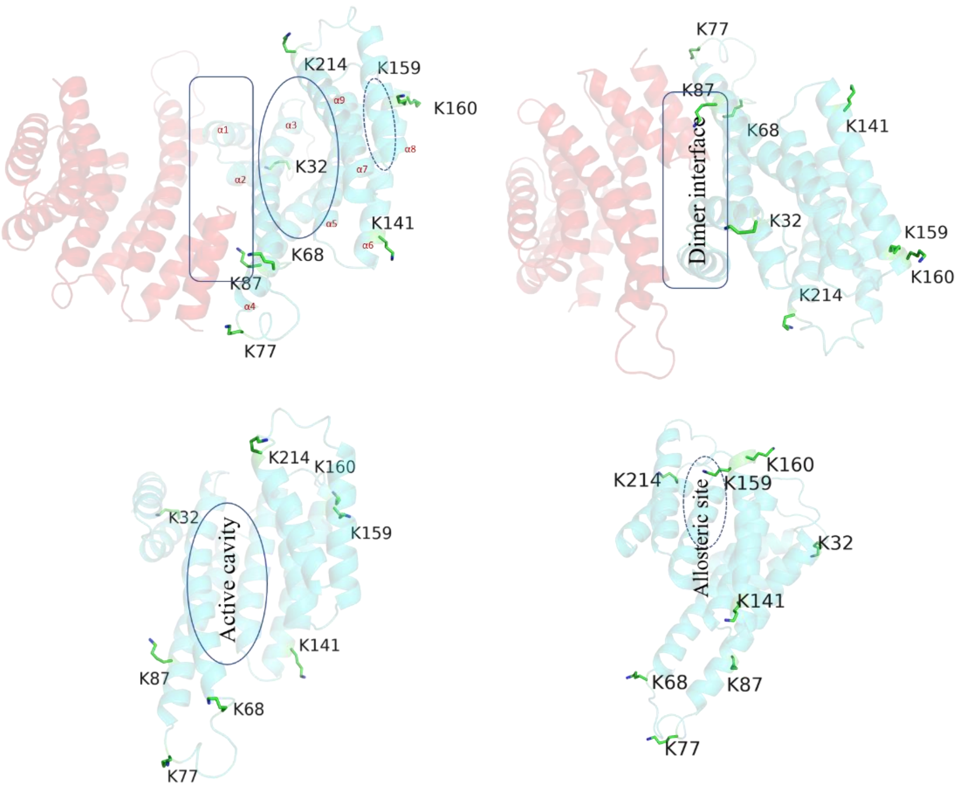

Figure S21. Sites of the lysine residues on 14-3-3σ with the active regions.

These lysine residues are plotted using stick style and colored in green. The A chain of 14-3-3σ is colored in blue, and the B chain of 14-3-3σ is colored in red. α-Helix of the 14-3-3σ monomer is labeled in red.

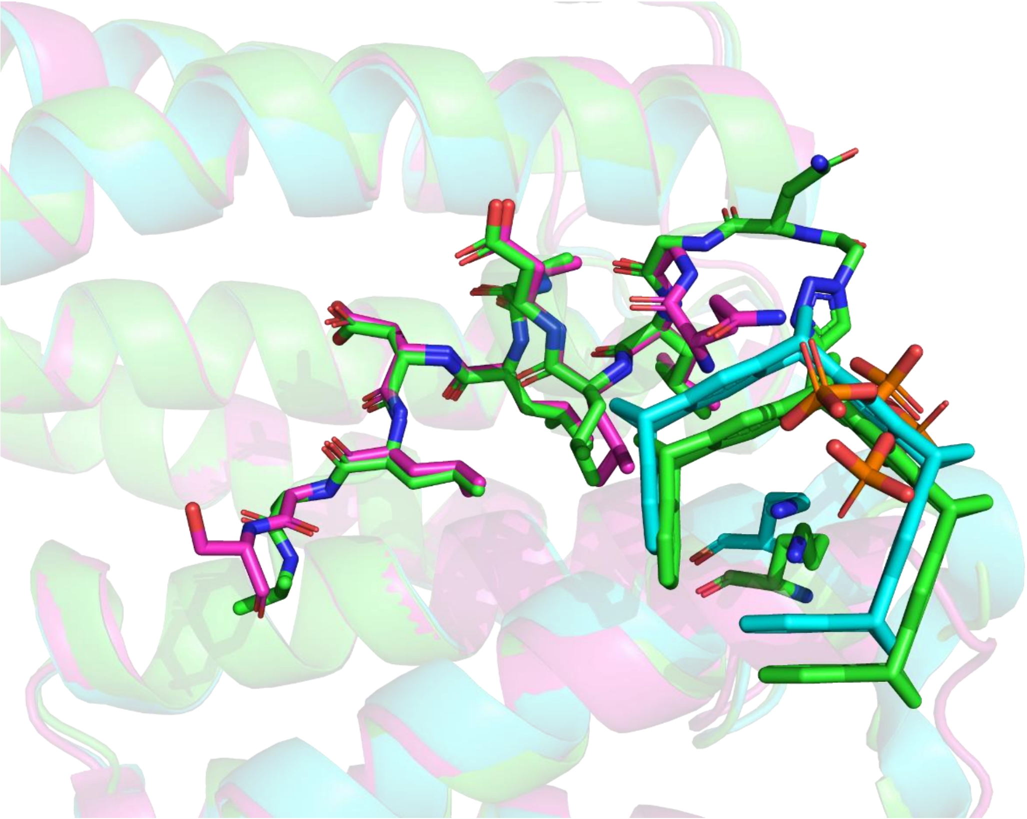

Figure S22. Superimposed structures of MT-ExoS, CLR01, ExoS binding to 14-3-3. The structure of the MT-ExoS bound with 14-3-3σ (PDB ID: 6Y7T) is colored with green, and the tweezer molecule and K214 are plotted by stick style. The structure of tweezer/K214 on 14-3-3σ (PDB ID: 5OEG) is colored with blue, and the tweezer molecule and K214 are plotted by stick style. The structure of the ExoS peptide bound with 14−3−3ζ (PDB ID: 4N7G) is colored with magenta, and the ExoS peptide is plotted by stick style.

Table S1. Simulation time of tweezer binding to lysine residues on 14-3-3σ for metadynamics.

Lysine Site	Time (ns)
K11	30
K27	30
K32	18
K49	62
K68	40
K77	30
K87	22
K109	30
K141	18
K159	40
K160	30
K195	30
K214	30

Table S2. Binding free energy (ΔG) and binding energy barrier for tweezer binding to lysine residues from metadynamics.

Type	Strong Binding Sites				Weak Binding Sites			
	K214	K77	K141	K160	K32	K68	K87	K159
ΔG	-10.5	-10.1	-10.3	-9.3	-5.7	-7.0	-7.5	-5.5
Binding Energy Barrier	3.7	1.3	2.6	3.2	3.3	3.8	1.8	2.1

Energies are in $kcal \cdot mol^{-1}$.

Table S3. Distance of the upward phosphate group (P1) of tweezer and the ammonium group of lysine residue in the bound state.

Lysine residue	D_{N-P} (Å)
K214	4.20±0.77
K77	4.05±0.51
K141	4.09±0.34
K160	4.09±1.12
K32	4.30±0.72
K68	4.36±0.84
K87	4.63±0.93
K159	4.45±0.78

D_{N-P} represents the distance between the P1 of tweezer and the amino group of lysine residue. This value consists of the average value and its standard deviation calculated from the all conformations in the bound state.
